# Rapid Dereplication of Bioactive Compounds in Plant and Food Extracts Using Liquid Chromatography–Electrospray–Tandem Mass Spectrometry

**DOI:** 10.1002/ansa.70038

**Published:** 2025-08-26

**Authors:** Naheed Akhtar, Adeeba Khadim, Syed Usama Yaseen Jeelani, Bibi Zareena, Arslan Ali, Jalal Uddin, Hesham R. El‐Seedi, Satyajit D. Sarker, Muhammad Ramzan, Syed Ghulam Musharraf

**Affiliations:** ^1^ H.E.J. Research Institute of Chemistry International Center for Chemical and Biological Sciences University of Karachi Karachi Pakistan; ^2^ Dr. Panjwani Center For Molecular Medicine and Drug Research International Center for Chemical and Biological Sciences University of Karachi Karachi Pakistan; ^3^ Department of Pharmaceutical Chemistry College of Pharmacy King Khalid University Asir Saudi Arabia; ^4^ Department of Chemistry Faculty of Science Islamic University of Madinah Madinah Saudi Arabia; ^5^ Centre For Natural Products Discovery School of Pharmacy and Biomolecular Sciences Faculty of Science Liverpool John Moores University Liverpool UK; ^6^ School of Chemistry and Pharmaceutical Sciences Guangxi Normal University Guilin China

**Keywords:** dereplication, flavonoids, LC–HR–ESI–MS/MS, tandem mass spectral library, triterpenes

## Abstract

High‐throughput screening and identification of common phytochemicals are crucial for lead optimization, drug development and investigation of metabolic pathways in complex herbal extracts. The available databases contain a huge number of compounds, making it challenging and time‐consuming to dereplicate valuable compounds. Therefore, the current study aimed to develop an in‐house mass spectral library for the rapid dereplication of 31 commonly occurring natural products from different classes using liquid chromatography–electrospray ionization–tandem mass spectrometry (LC–ESI–MS/MS). A total of 31 standards were grouped into two different pools, and each pool was analysed under uniformly optimized conditions in positive ionization mode. A pooling strategy on the basis of log *P* values and exact masses was adopted to minimize the co‐elution and the presence of isomers in the same pool. The MS/MS features of each compound were acquired using [M + H]^+^ and/or [M + Na]^+^ adducts at 25.5–62 eV range as average collision energy and 10, 20, 30 and 40 eV as individual collision energies. The names, molecular formulae, exact masses with <5 ppm error, MS and MS/MS features of analysed reference compounds were used to construct the MS/MS library. The developed MS/MS library was efficiently used for the rapid dereplication and validation of 31 compounds in 15 different food and plant sample extracts. The MS data of 31 reference standards have been submitted to the MetaboLights online database (MTBLS9587). The developed library will be beneficial for the rapid dereplication of biologically valuable compounds in a variety of herbal formulations and food samples.

## 1 Introduction

Plants produce a unique chemical, biological, and structural diversity of natural products [1]. According to Newman and Cragg, from 1981 to 2019, half of the newly approved drugs were obtained from medicinal plants [2]. Medicinal plants have received special attention for identifying the new bioactive molecules that may be used to treat a wide range of diseases, including cardiovascular disease, Parkinson's disease, hepatitis, HIV/AIDS, diabetes, inflammatory diseases and Alzheimer's disease, and so on [3]. Medicinal plants such as flavonoids, phenolic acids, stilbenoids, coumarin, triterpenes and sterols that exhibit distinctive biological activities such as anti‐cancer, anti‐diabetic [4], anti‐inflammatory, anti‐viral, anti‐bacterial, antioxidant, anti‐fungal [5, 6], anti‐tumour, anti‐microbial [7], anti‐tubercular, anti‐coagulant, anti‐hypertensive [8], anti‐carcinogenic and cholesterol‐lowering activity [9]. These compounds also demonstrate strong hepatoprotective [10], cardioprotective, neuroprotective [11] and anti‐pyretic properties.

The careful isolation and characterization of natural products in complex plant extracts or herbal formulations remains a major challenge in the search for new lead compounds. The rediscovery of known compounds is a common outcome following labour‐intensive and time‐consuming chromatographic and isolation procedures, making the identification of novel constituents particularly difficult.

Furthermore, the usage of herbal medicinal products is expanding globally. Although some herbal products are widely used, many remain untested due to incomplete identification of active constituents and limited knowledge of their potential adverse effects. Manufacturers of herbal medicinal products also face considerable difficulties in establishing chemical standardization protocols, largely due to the immense diversity of plant‐derived natural products [12]. Comprehensive identification of active constituents, along with an understanding of their pharmacological effects and possible toxicities, is essential to ensure the consistency and reproducibility of pharmacological and clinical studies, as well as to maintain the quality and efficacy of herbal products [13].

The dereplication strategy offers valuable structural information and prevents the isolation and re‐characterization of well‐known compounds [14]. Several analytical methods, such as spectroscopic and mass spectrometric methods, have been employed for the determination, characterization and quality control of natural products in crude plant extracts [15]. Several mass spectral databases and libraries are available free of cost for the characterization of several natural products in botanical extracts, such as NIST [16], WEIZMASS [17], MassBank [16], mzCloud [18], MassBank of North America (MoNA) [16], Global Natural Products Social (GNPS) molecular networking [19], RIKEN tandem mass spectral database (ReSpect) [20], HMDB [16, 21] and METLEN [16, 22]. Although the above‐mentioned dereplication methods or databases are efficient and include useful information about the secondary metabolites, they have some limitations. Most of them are based on the analysis of individual compounds and often lack comprehensive mass spectral data of both [M + H]^+^ and [M + Na]^+^ ions. Some databases do not contain information about retention times (RT) and chromatographic profiles, which improve confidence in metabolite annotation, because they are based on direct infusion MS/MS data. Some databases, such as MoNa and NIST‐14 library, include chromatographic features but do not provide visual representations of precursor ion chromatographic peaks [23]. Moreover, these databases typically include several thousand compounds, which require considerable time and effort to search and screen for the compound of interest. Therefore, there is a need to develop a rapid, simple and cost‐effective mass spectral database that includes both chromatographic and MS/MS data to support the quick dereplication of phytochemicals in food and plant extracts. In the current study, a rapid and sensitive data processing strategy was employed for the dereplication of 31 biologically significant phytochemicals through the development of a high‐resolution tandem mass spectral library. Additionally, the use of a pooling strategy in combination with relative RT makes the current study a cost‐effective and time‐saving approach compared to an individual analysis of the reference compounds, as commonly reported in previous studies. The developed library includes chromatographic peaks and MS/MS spectral data of both [M + H]^+^ and [M + Na]^+^ adducts of the studied compounds. Different food and plant extracts were screened and validated against the developed database. The developed database provides a novel and efficient method for the rapid dereplication and identification of common secondary metabolites in a variety of samples, including food and plant extracts.

## 2 Experimental Procedures

### 2.1 Chemicals and Reagents

Analytical grade chemicals, solvents and standards were used in the current analysis. Type‐1 water (ISO 3696, resistivity: 18.1 M Ω cm at 25°C) was obtained from an ultrapure water purification system (Branstead GenPure, Waltham, MA, USA) and used as a mobile phase. Methanol (MeOH) and formic acid (mobile phase additive) were purchased from Merck (Merck KGaA, Darmstadt, Germany) and Dae‐Jung (Dae‐Jung Chemicals & Metals Co. Ltd., Korea), respectively. Thirty‐one standard compounds (purity 97%–98%) were obtained from Sigma‐Aldrich (USA). The names, molecular formulas, classes, structures and log *P* values of analysed standards are displayed in Table 1.

**TABLE 1:** List of standards used in the library development of pools 1 and 2.

Pool‐1										
S. no.	Compound name	Log *P* value	RT (min)	Mol. formula	Calculated mass	Observed masses	Error (ppm)	Ion type	Class of compound	MS/MS
1.	Quercetin	2.07	4.34	C_15_H_10_O_7_	325.0318	325.0327	2.77	[M + Na]^+^	Flavonol	325.0327
2.	Catechin	0.49	4.65	C_15_H_14_O_6_	313.0680	313.0676	−0.32	[M + Na]^+^	Flavonoid	313.0699, 225.1029
3.	Chlorogenic acid	−0.36	4.74	C_16_H_18_O_9_	377.0843	377.0834	−2.39	[M + Na]^+^	Phenolic acid	377.0834, 316.3498
4.	Rutin	1.76	5.89	C_27_H_30_O_16_	633.1426	633.1435	1.42	[M + Na]^+^	Flavonol	633.1488, 331.0998
5.	Isorhamnetin	1.76	7.31	C_16_H_12_O_7_	339.0475	339.0473	−0.59	[M + Na]^+^	Flavonol	339.0473, 321.0369
6.	Diosmetin	3.1	7.46	C_16_H_12_O_6_	323.0526	323.0510	−4.95	[M + Na]^+^	Flavone	323.0513, 281.9245, 238.8999
7.	*Trans*‐ferulic acid	1.64	6.06	C_10_H_10_O_4_	217.0471	217.0479	3.69	[M + Na]^+^	Phenolic acid	217.0479, 172.9765
8.	Myricetin	2.11	7.94	C_15_H_10_O_8_	319.0448	319.0450	0.63	[M + H]^+^	Flavonol	319.0455, 225.0545, 197.0587, 150.0438
9.	Apigenin	2.1	8.18	C_15_H_10_O_5_	271.0601	271.0591	−3.69	[M + H]^+^	Flavone	271.0596, 178.9209, 153.0180
10.	Cinnamic acid	2.41	5.46	C_9_H_8_O_2_	171.0417	171.0410	−4.09	[M + Na]^+^	Phenolic acid	171.0410, 153.0189, 138.9913
11.	Myricitrin	1.98	5.9	C_21_H_20_O_12_	487.0846	487.0832	−2.87	[M + Na]^+^	Flavonol	487.0846, 425.2923, 372.9568, 323.0190
12.	Betulinic acid	8.94	10.57	C_30_H_48_O_3_	479.3495	479.3488	−1.46	[M + Na]^+^	Triterpene	479.3496, 435.3624, 219.1730
13.	Friedelin	10.87	11.67	C_30_H_50_O	427.3934	427.3942	1.87	[M + H]^+^	Triterpene	427.3942, 401.2333, 383.2245, 342.1973
14.	Betulin	9.01	11.05	C_30_H_50_O_2_	465.3703	465.3713	2.15	[M + Na]^+^	Triterpene	465.3717, 376.2967, 295.1942
15.	Stigmasterol	10.21	11.09	C_29_H_48_O	413.3777	413.3780	0.73	[M + H]^+^	Sterol	413.3780, 301.1427, 189.0146

Pool‐2										
1.	Isoquercitrin	1.75	5.78	C_21_H_20_O_12_	487.0846	487.0844	−0.41	[M + Na]^+^	Flavonol	487.0838, 341.0280
2.	Kaempferide (Kaempferol 4′‐*O*‐methyl ether)	2.74	7.46	C_16_H_12_O_6_	323.0526	323.0513	−4.02	[M + Na]^+^	Flavonol	323.0513, 281.9245, 238.8999
3.	Chrysin	2.88	8.90	C_15_H_10_O_4_	255.0652	255.0644	−3.14	[M + H]^+^	Flavone	255.0644, 153.0176
4.	*Trans*‐resveratrol	3.14	8.28	C_14_H_12_O_3_	229.0859	229.0858	−0.44	[M + H]^+^	Stilbenenoid	229.0859, 211.0752, 183.0389, 175.0388
5.	Hesperidin	1.78	6.07	C_28_H_34_O_15_	611.1970	611.1967	−0.49	[M + H]^+^	Flavanones	611.1973, 449.1435, 303.0866
6.	Hesperetin	2.9	7.06	C_16_H_14_O_6_	325.0682	325.0681	−0.31	[M + Na]^+^	Flavanone	325.0681, 280.8812, 268.3916, 225.9143
7.	Naringenin	3.19	6.92	C_15_H_12_O_5_	273.0757	273.0761	1.46	[M + H]^+^	Flavanone	273.0758, 153.0184
8.	Kaempferol	2.05	7.17	C_15_H_10_O_6_	287.0550	287.0544	2.09	[M + H]^+^	Flavonol	287.0557, 243.0566, 203.0334, 153.0189, 129.0607
9.	Galangin	2.83	8.17	C_15_H_10_O_5_	271.0601	271.0602	0.37	[M + H]^+^	Flavonol	271.0603, 243.0632, 153.0182
10.	Herniarin	1.78	5.79	C_10_H_8_O_3_	199.0365	199.0357	−4.02	[M + Na]^+^	Coumarin	199.0357, 155.0029
11.	Hederagenin	7.41	10.11	C_30_H_48_O_4_	495.3444	495.3440	−0.81	[M + Na]^+^	Triterpene	495.3452, 461.2859, 439.0354, 387.7562
12.	Oleanolic acid	9.06	10.56	C_30_H_48_O_3_	479.3495	479.3496	0.21	[M + Na]^+^	Triterpenoid	479.3496, 433.3624, 195.9766
13.	β‐sitosterol	10.73	9.42	C_29_H_50_O	437.3754	437.3760	1.37	[M + Na]^+^	Sterol	437.3760
14.	Maslinic acid	7.87	10.16	C_30_H_48_O_4_	495.3444	495.3441	−0.61	[M + Na]^+^	Triterpene	495.3456, 450.2247, 288.9233, 226.9509
15.	Lupeol	10.98	11.68	C_30_H_50_O	427.3934	427.3942	1.87	[M + H]^+^	Triterpenoid	427.3941, 401.2333, 383.2245, 342.1973
16.	Betulonic acid	8.36	10.80	C_30_H_46_O_3_	477.3339	477.3332	0.21	[M + Na]^+^	Triterpene	477.3340, 433.3441, 406.3266, 317.1036

Abbreviation: RT, retention times.

### 2.2 Preparation of Standard Solutions and Sample Extracts

1 mg of each standard compound was dissolved in 1 mL of methanol for the preparation of the standard stock solution and kept below 0°C. Two pools were prepared from standard stock solutions on the basis of the exact masses and log *P* values of the standard compounds, which were calculated using ChemSketch ver. 2081.1 (ACD/Lab, Inc.). The pools were prepared by mixing 10 µL of 15 and 16 standard compounds individually. The pooled samples were diluted (100 and 1000 times), resulting in the final concentrations of 100 and 10 µg/mL, respectively. The resulting solutions were filtered through a Millipore syringe filter (0.22 µm) prior to LC–MS/MS analysis.

A total of 15 different plants and food samples were used in the current study. Six plant species were collected from different regions of Pakistan, including Karachi, Islamabad, Neelum Valley and Azad Kashmir, and identified by a plant taxonomist. The methanolic extracts of plants were prepared by accurately weighing one gram of powder material soaked in 10 mL of methanol and subjected to sonication at room temperature for 15–20 min. After sonication, the samples were centrifuged at 6000 rpm for 30 min, and the resultant supernatants were filtered through a 0.22 µm PTFE syringe filter. A volume of 5 mL of each sample was taken and dried in a vacuum concentrator, then 2 mg of dried sample was dissolved in 1 mL HPLC grade MeOH in HPLC vials and stored in a refrigerator for liquid chromatography–electrospray ionization–tandem mass spectrometry (LC–ESI–MS/MS) analysis.

Food samples, including tomato, potato, spinach, apple, strawberry, banana, rice, almond and cashew, were purchased from local markets and supermarkets in Karachi, Pakistan. All food samples were crushed and homogenized in a mortar using liquid nitrogen. Ten grams of each powdered sample were extracted with 10 mL of methanol and centrifuged at 12,000 rpm for 5 min to settle down the solid residue. The supernatants were filtered using 0.22 µm PTFE syringe filters. A volume of 5 mL of each food sample was taken and dried in a vacuum concentrator, then 2 mg of the dried sample was dissolved in 1 mL of methanol. After filtration, the prepared samples were transferred into HPLC vials and subjected to LC–ESI–MS/MS analysis [24]. The final concentration of each extract was 2 mg/mL.

### 2.3 Instrumentation and Experimental Conditions

The analysis was performed using Bruker's MaXis‐II ESI‐QTOF‐MS instrument (Bruker Daltonics, Bremen, Germany), equipped with Dionex UltiMate 3000 series ultra‐high‐performance liquid chromatography (UHPLC) system (Thermo Fisher Scientific, Waltham, MA, USA) with an autosampler, thermostated column compartment, degasser and a binary pump. A volume of 10 mM sodium formate solution was injected through a syringe pump as an internal calibrant for mass scale calibration at a flow rate of 3 µL/min. A reverse‐phase Macherey‐Nagel NUCLEODUR C18 gravity column (2.0 mm × 100 mm, 1.8 mm) with a guard column (2.0 mm × 4 mm, 1.8 mm) was used. The mobile phase comprises solvent A (0.1% formic acid in deionized water) and solvent B (0.1% formic acid in methanol). To remove dissolved gases, mobile phases were sonicated for 15 min. The injection volume of each pool was set at 5 µL with a flow rate of 0.5 mL/min. The linear mobile phase gradient was set as 5% B was maintained for 1 min, then a linear gradient of 5%–95% B in 1–9 min, 95% B for 9–10 min, 5% B for 10.7–13 min was employed for the sample elution. The total run time of the analysis of each sample was 13 min, with a post‐run of 1 min. A volume of 10 mM solution of ammonium formate and 0.1 mM solution of sodium chloride were added in both mobile phases A and B to obtain ammonium and sodium adducts, respectively. The analysis was done in positive electrospray ionization mode. The following parameters were used in the current analysis: capillary voltage at 4500 V, end plate offset at 500 V, nebulizer gas N_2_ pressure at 2.8 bar, drying gas (N_2_) at 10.01 mL/min and drying gas temperature at 300°C. A scan speed of 5 Hz for MS and 12 Hz for MS/MS was used to record MS/MS spectra in the mass range of 90–2000 *m/z* [25]. The recorded MS and MS/MS data were analysed through Bruker Compass DataAnalysis (version 4.4) and then added into Bruker Library Editor (4.4) for MS/MS library development and identification of reference compounds in different matrices.

### 2.4 Data Analysis

The mass spectra of each standard compound from two different pools were acquired using target analysis and extracted ion chromatogram (EIC). The targeted screening of the obtained data was performed using Bruker Compass Target Analysis software version 1.3 (Bruker Daltonics) by generating a comma‐delimited value list (CSV file) and a search list for each pool of standards separately. This file contained information about each compound's potential sodium adducts as well as RT, *m/z*, names and chemical formulas under 5 ppm error and mSigma value <50. Bruker Compass DataAnalysis (version 4.4) software was used for further analysis of processed targeted data files for acquiring mass spectral data of each reference compound. All the MS and MS/MS features of the analysed reference compounds were collected in the form of a mass spectral library using Library Editor 4.4 (Bruker). Similarly, the data of plant extracts was calibrated and screened against the developed library for the identification of reference compounds in different matrices.

## 3 Results and Discussion

### 3.1 LC–MS Analysis and MS/MS Library Development

**FIGURE 1:**
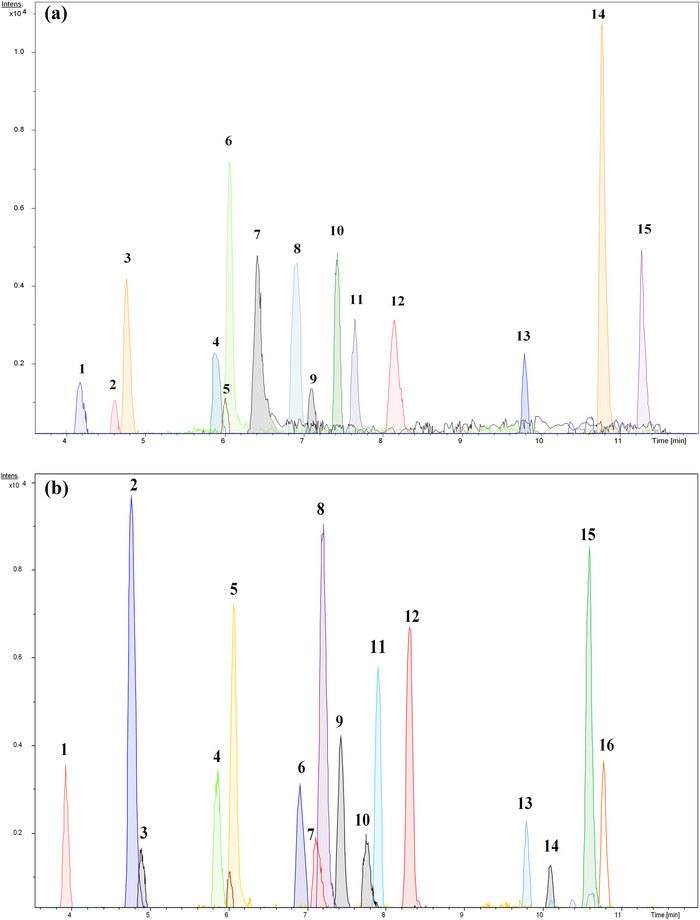
Extracted ion chromatograms (EICs) of reference standards, pool‐1 (a) and pool 2 (b).

In this study, 31 standard compounds were grouped into 2 pools on the basis of their log *P* values. This pooling strategy was adopted to prevent the co‐elution of compounds with similar or very close log *P* values, which often give similar polarity and lead to the overlapping of RT during LC separation. Additionally, the compounds with similar or very close log *P* values and exact masses were kept in separate pools to minimize the chance of isomers being present in the same pool. A total of 2 pools were prepared; pool‐1 consists of 15 compounds, whereas pool‐2 consists of 16 compounds. For the separation of compounds, the LC method was developed using RP‐C18 under a linear gradient. The total run time of analysis was 13 min, and all the compounds were eluted successfully within the analysis time. Figure 1 shows the EICs of reference standards, pool‐1 (A) and pool‐2 (B). The reference compounds in both pools were detected on the basis of RT, exact masses with mass accuracy with <5 ppm error, and fragmentation patterns of each compound. Table 1 displays the names, molecular formula, log *P* values and detection parameters of the compounds in each pool.

**FIGURE 2:**
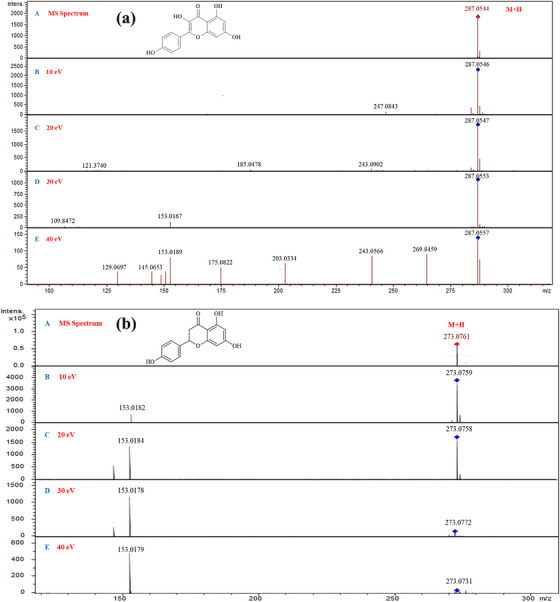
MS and MS^2^ spectra of (a) kaempferol and (b) naringenin: (A) MS, (B) 10 eV, (C) 20 eV, (D) 30 eV and (E) 40 eV.

Two acquisition methods, auto MS/MS and targeted MS/MS analysis, were performed in a separate analytical run and used to obtain the tandem mass spectral features of reference compounds. For auto MS/MS or data‐dependent acquisition (DDA) analysis, all the reference compounds were analysed using collision energies in the range of 25.5–62 eV. The acquisition method provides information about the RT, MS and MS/MS features of the studied compounds. The targeted MS/MS analysis of reference compounds was achieved using the scheduled precursor list (SPL) of each compound that possesses the four different collision energies, including 10, 20, 30 and 40 eV. In response to the applied collision energies, the reference compounds were fragmented in the QTOF collision cell and produced a characteristic MS/MS pattern. So, this acquisition method provides the information about MS/MS spectra of specific collision energy using RT. Figures S1–S31 depict the MS/MS spectra and chemical structures of reference compounds from each pool. Kaempferol showed the highest intensity peak as [M + H]^+^ ion with *m/z* 287.0544 at 10 eV, which declines significantly at 20, 30 and 40 eV, with the increasing number of fragment ions at 40 eV. Figure 2 shows the MS and MS^2^ spectra of (a) kaempferol and (b) naringenin at four different collision energies of 10, 20, 30 and 40 eV. In this analysis, the analysed compounds yield enough fragmentation in response to the applied collision energies. The resulting fragmentation pattern showed that most of the analysed compounds produced [M + H]^+^ and [M + Na]^+^ adducts. All the chromatographic and MS/MS spectral information of analysed compounds were collected and compiled as a mass spectral library using Bruker Compass Library Editor (4.4) [26, 27]. The mass spectral data of 31 reference compounds have been submitted to the MetaboLights online database (MTBLS9587). Figure S32 depicts the kaempferol library record in Bruker Library Editor 4.4. The developed database can be beneficial for the rapid and accurate identification of compounds using both proton and sodium adducts when similar compounds are present in a complex mixture.

### 3.2 Tandem Mass Spectral Features of Reference Standards

The current study involves the analysis of different classes of compounds such as terpenes, polyphenols, sterols, coumarins and stilbenoids. The analysed compounds were fragmented in the QTOF collision cell and produced characteristic MS/MS structural features. The most common fragments observed in polyphenols are typically explained by the neutral losses of H_2_O and CO_2_. In positive ionization mode, flavanols showed a loss of H_2_O molecules due to the presence of a hydroxyl group on the central ring C, resulting in the ring A or ring B being retained during the fragmentation process. Flavonoids were also identified by the fragmentation of compounds undergoing retro‐Diels–Alder (RDA) reaction [28]. For example, in our study, naringenin was observed as [M + H]^+^ at *m/z* 273.0761, which undergoes RDA fragmentation and gives rise to the formation of product ion at *m/z* 153.0184 as reported by Cavaliere et al. [29]. In positive ionization mode, coumarins undergo sequential fragmentation and usually form a protonated molecular ion as [M + H]^+^ and a sodium adduct as [M + Na]^+^ [30]. In coumarins, the fragmentation is affected by the heterocyclic ring structure and substituent on the basic coumarin skeleton [31]. Due to the basic lactone ring system in the structure, they are inclined to give a neutral loss of CO_2_ [32] and CO [33]. In this case, herniarin (7‐methoxy coumarin) forms a sodiated adduct [M + Na]^+^ at *m/z* 199.0357 and undergoes a neutral loss of CO_2_ molecule with *m/z* 155.0029. In ESI mode, the fragmentation pattern of pentacyclic triterpenes depends on the specific groups attached to the basic skeleton, such as carboxylic moieties, position and number of double bonds and hydroxyl groups. However, fragmentation occurs as the result of single or multiple removal of substituents like CH_3_ and H_2_O from the protonated molecular ion, and a C‐ring cleavage by RDA reaction provides the most common and important fragmentation information for pentacyclic triterpenes [34]. In our case, oleanolic acid [M + Na]^+^ at *m/z* 479.3496 showed a fragment ion at *m/z* 433.3624 as the loss of a HCOOH molecule as reported by Pham et al. [35]. One of the most common fragmentation pathways in stilbenoids involves the neutral loss of the H_2_O molecule, CO, and the cleavage of a carbon–carbon bond in one of the phenyl rings of the basic stilbene structure [36]. In our study, we observed resveratrol at *m/z* 229.0858 as [M + H]^+^, and two characteristic fragment ions at *m/z* 211.0752 and 183.0389 were observed as a loss of H_2_O and CO molecules in trans‐resveratrol as reported by Zhan et al. [37]. Figure S33 depicts the common fragmentation pathway of the investigated analyte.

### 3.3 Screening of Chemically Diverse Food and Plant Extracts

Fifteen different food and plant extracts were screened against the developed library for the rapid identification of pharmacologically important metabolites of plant origin using standardized search parameters. Different features were generated for the investigation of sample extracts using the MS(*n*) tool of all analysed compounds, including EICs, exact masses, RT and MS/MS fragments.

**FIGURE 3:**
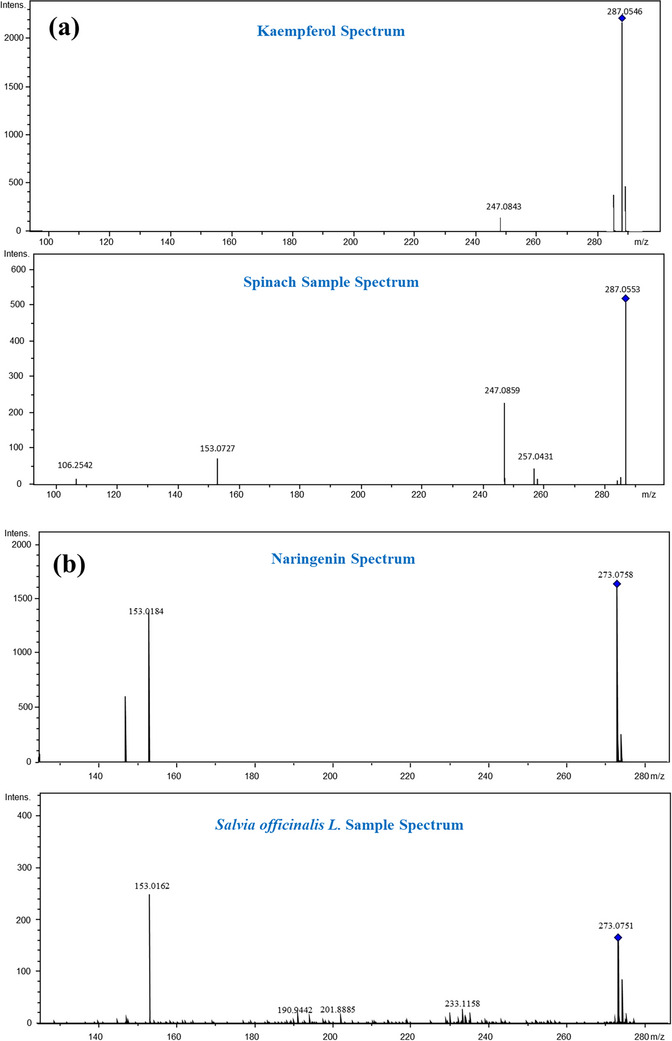
MS^2^ spectrum of kaempferol (a) naringenin (b) from the Library Editor 4.4 (top) and real samples (bottom).

The identified compounds in each food and plant extract against the developed library are listed in Table 2. The compounds were identified on the basis of their fragmentation behaviour, exact masses (mass tolerance window, ±0.005 Da), and RT in comparison with the standard compounds existing in the spectral library. All the analysed compounds achieved the identification level‐01 and level‐03 as described by AC Schrimpe‐Rutledge et al. [38]. Figure 3 shows the MS^2^ spectrum of kaempferol (a) and naringenin (b) from the Library Editor 4.4 (top) and spinach and *Salvia officinalis* L. samples (bottom), respectively.

**TABLE 2:** Identified compounds in food and plant extracts using a developed phytostandard library.

S. no.	Source	Compound name	RT (min)	Drift RT (min)	Fragments
Plant samples					
1	*Salvia officinalis L*	Naringenin	7.22	+0.3	273.0758, 153.0184
		Lupeol	11.48	−0.2	427.3941, 401.2333, 383.2245, 342.1973
2	*Ziziphus jujuba*	Apigenin	7.77	−0.41	271.0596, 178.9209, 153.0180
3	*Aquilegia fragrans*	Quercetin	4.4	+0.06	325.0327
4	*Anemone falconeri*	Lupeol	11.48	−0.2	4.27.3941, 401.2333, 383.2245, 342.1973
		Stigmasterol	11.19	+0.1	413.3780, 301.1427, 189.0146
5	*Anemone obtusiloba*	Isoquercitrin	5.75	−0.03	487.0838, 341.0280
6	*Cestrum diurnum*	Betulonic acid	10.75	−0.05	477.3340, 433.3441, 406.3266, 317.1036
		Friedelin	11.7	−0.03	427.3942, 401.2333, 383.2245, 342.1973

Food samples					
7	Tomato	Rutin	5.97	+0.08	633.1488, 331.0998
8	Potato	Kaempferol	7.37	+0.2	287.0557, 243.0566, 203.0334, 153.0189, 129.0607
		Apigenin	7.77	−0.41	271.0596, 178.9209, 153.0180
		Chlorogenic acid	4.66	−0.08	377.0834, 316.3498
9	Spinach	Quercetin	4.4	+0.06	325.0327
		Kaempferol	7.37	+0.2	287.0557, 243.0566, 203.0334, 153.0189, 129.0607
		Apigenin	7.77	−0.41	271.0596, 178.9209, 153.0180
10	Apple	Apigenin	7.77	−0.41	271.0596, 178.9209, 153.0180
		Herniarin	4.89	−0.9	199.0357, 155.0029
		Oleanolic acid	10.36	−0.2	479.3496, 433.3624, 195.9766
11	Strawberry	β‐sitosterol	9.44	+0.02	437.3760
		Chlorogenic acid	4.66	−0.08	377.0834, 316.3498
12	Banana	Stigmasterol	11.19	+0.1	413.3780, 301.1427, 189.0146
13	Rice	Myricetin	7.9	−0.04	319.0455, 225.0545, 197.0587, 150.0438
		Catechin	4.64	−0.01	313.0699, 225.1029
14	Almond	Kaempferol	7.37	+0.2	287.0557, 243.0566, 203.0334, 153.0189, 129.0607
		Catechin	4.64	−0.01	313.0699, 225.1029
		Quercetin	4.4	+0.06	325.0327
15	Cashew	Myricetin	7.9	−0.04	319.0455, 225.0545, 197.0587, 150.0438
		Quercetin	4.4	+0.06	325.0327
		Kaempferol	7.37	+0.2	287.0557, 243.0566, 203.0334, 153.0189, 129.0607

Abbreviation: RT, retention times.

### 3.4 Comparison With Other Methods

Several LC–MS/MS studies have been conducted so far for the analysis of a variety of phytochemicals. Some studies related to the qualitative analysis, whereas others involved the quantitative analysis of common metabolites of different classes. For example, Haq et al. reported an LC–MS/MS‐based method for the dereplication of *Salvia* species [39]. Elmaidomy et al. documented LC–HR–ESI–MS‐based dereplication of *Premna odorata* leaves [40]. Razali et al. reported LC–MS‐Orbitrap metabolite profiling and fingerprinting of *Zingiber zerumbet* (L.) Roscoe ex Sm [41]. Ayoka et al. reported the UHPLC–ESI–QTOF MS/MS method for the investigation of potential anti‐oxidants in *Zanthoxylum zanthoxyloides* leaves [42]. Sahu et al. reported an LC–MS–DNP‐based dereplication of *Araucaria cunninghamii* Mudie gum‐resin [43]. All these methods do not involve any library generation or pooling of compounds on the basis of the log *P* values. Few studies have provided these characteristics, but all these databases are related to specific classes of compounds or drugs. For example, Zareena et al. reported a library of 44 triterpenoids [44]. Aziz et al. reported a mass spectral library of 40 flavonoids [25]. Zareena et al. reported a mass spectral library of 161 alkaloids [27]. Khadim et al. reported a mass spectral database of 491 pharmaceutical drugs [26]. Compared to the existing literature, the current work presents a significant advancement by the development of an in‐house mass spectral library of 31 prevalent phytochemicals from various structural classes. A key advantage of this study is its ability to enable the screening and dereplication of multiple classes of compounds in a wide range of herbal extracts without the need to reanalyse individual reference compounds. Unlike several previously reported methods, the developed library integrates both chromatographic and mass spectral features, which are essential for the accurate identification of common metabolites in complex mixtures.

## 4 Conclusions

A robust and sensitive strategy for the dereplication of biologically important common phytochemicals has been successfully implemented through the development of an LC–ESI–QTOF–MS/MS‐based spectral library. A total of 31 biologically important compounds were pooled into two pools on the basis of log *P* values and analysed in the positive ionization mode of MS. The employed pooling strategy proved efficient in terms of reducing time, cost and co‐elution of compounds with similar polarities. The detection of reference compounds was achieved on the basis of RT, exact masses with mass accuracy <5 ppm error and fragmentation patterns for the development of a high‐resolution mass spectral library. The developed MS/MS library was also successfully applied for the detection and identification of reference compounds in 15 different plant extracts and food samples. This study demonstrates the utility of the approach for the rapid dereplication and identification of biologically valuable compounds in different food extracts, herbal formulations and complex plant extracts. Such strategies may be further developed and implemented both on small and large scales for the detection and traceability of several compounds to monitor the overall quality of foods, plant extracts and different herbal formulations.

## Author Contributions

**Naheed Akhtar**: conceptualization, methodology, validation, investigation, writing – original draft preparation, project administration. **Adeeba Khadim**: methodology, data curation, writing – original draft preparation. **Syed Usama Yaseen Jeelani**: software, data curation, writing – original draft preparation. **Bibi Zareena**: software, investigation. **Arslan Ali**: validation, data curation. **Jalal Uddin**: formal analysis, writing – review and editing, visualization. **Hesham R. El‐Seedi**: validation, writing – review and editing. **Satyajit D. Sarker**: formal analysis, writing – review and editing. **Muhammad Ramzan**: formal analysis. **Syed Ghulam Musharraf**: conceptualization, resources, writing – review and editing, supervision funding acquisition. All authors have read and agreed to the published version of the manuscript.

## Conflicts of Interest

The authors declare no conflicts of interest.

## Supporting information




**Supporting File 1**: ansa70038‐sup‐0001‐SuppMat.pdf

## Data Availability

The data that support the findings of this study are available from the corresponding author upon reasonable request.
