# Supplementary material for: Rapid Dereplication of Bioactive Compounds in Plant and Food Extracts Using Liquid Chromatography–Electrospray–Tandem Mass Spectrometry
Source: Anal Sci Adv. 2025 Aug 26;6(2):e70038. doi: 10.1002/ansa.70038 (PMC12380011; doi:10.1002/ansa.70038)
Supplement: Supplementary file 1 — Supporting File 1: ansa70038‐sup‐0001‐SuppMat.pdf [file ANSA-6-e70038-s001.pdf]

Rapid Dereplication of Bioactive Compounds in Plant and Food Extracts using Liquid Chromatography-Electrospray-Tandem Mass Spectrometry

Naheed Akhtar[a], Adeeba Khadim[a], Syed Usama Yaseen Jeelani[a], Bibi Zareena[a], Arslan Ali[b], Jalal Uddin[c], Hesham R. El-Seedi[d], Satyajit D. Sarker[e], Muhammad Ramzan[a], Syed Ghulam Musharraf[a,b,f,*]

[a] *H.E.J. Research Institute of Chemistry, International Center for Chemical and Biological Sciences, University of Karachi, Karachi, 75270, Pakistan;*

[b] *Dr. Panjwani Center for Molecular Medicine and Drug Research, International Center for Chemical and Biological Sciences, University of Karachi, Karachi, 75270, Pakistan;*

[c] *Department of Pharmaceutical Chemistry, College of Pharmacy, King Khalid University, Asir 61421, Saudi Arabia.*

[d] *Department of Chemistry, Faculty of Science, Islamic University of Madinah, Madinah 42351, Saudi Arabia*

[e] *Centre for Natural Products Discovery, School of Pharmacy and Biomolecular Sciences, Faculty of Science, Liverpool John Moores University, Byrom Street, L3 3AF, Liverpool, United Kingdom;*

[f] *School of Chemistry and Pharmaceutical Sciences, Guangxi Normal University, China*

** Corresponding author.*

Prof. Dr. Syed Ghulam Musharraf

H.E.J. Research Institute of Chemistry, International Center for Chemical and Biological Sciences, University of Karachi, Karachi-75270, Pakistan

Tel.: +92 213 4824924-5; 4819010; fax: + 92 213 4819018-9.

E-mail address: musharraf1977@yahoo.com

Supplementary Material

List of Supplementary Figures

SUPPLEMENTARY FIGURE. S1. MS spectrum of quercetin.

SUPPLEMENTARY FIGURE. S2. MS spectrum of catechin.

SUPPLEMENTARY FIGURE. S3 MS spectrum of chlorogenic acid.

SUPPLEMENTARY FIGURE. S4. MS spectrum of rutin.

SUPPLEMENTARY FIGURE. S5. MS spectrum of isorhamnetin.

SUPPLEMENTARY FIGURE. S6. MS spectrum of diosmetin.

SUPPLEMENTARY FIGURE. S7. MS spectrum of *trans*-ferulic acid.

SUPPLEMENTARY FIGURE. S8. MS spectrum of myricetin.

SUPPLEMENTARY FIGURE. S9. MS spectrum of apigenin.

SUPPLEMENTARY FIGURE. S10. MS spectrum of cinnamic acid.

SUPPLEMENTARY FIGURE. S11. MS spectrum of myricitrin.

SUPPLEMENTARY FIGURE. S12. MS spectrum of betulinic acid.

SUPPLEMENTARY FIGURE. S13. MS spectrum of friedelin.

SUPPLEMENTARY FIGURE. S14. MS spectrum of betulin.

SUPPLEMENTARY FIGURE. S15. MS spectrum of stigmasterol.

SUPPLEMENTARY FIGURE. S16. MS spectrum of isoquercitrin.

SUPPLEMENTARY FIGURE. S17. MS spectrum of kaempferide (kaempferol 4'-O-methyl ether).

SUPPLEMENTARY FIGURE. S18. MS spectrum of chrysin.

SUPPLEMENTARY FIGURE. S19. MS spectrum of *trans*-resveratrol.

SUPPLEMENTARY FIGURE. S20. MS spectrum of hesperidin.

SUPPLEMENTARY FIGURE. S21. MS spectrum of hesperetin.

SUPPLEMENTARY FIGURE. S22. MS spectrum of naringenin.

SUPPLEMENTARY FIGURE. S23. MS spectrum of kaempferol.

SUPPLEMENTARY FIGURE. S24. MS spectrum of galangin.

SUPPLEMENTARY FIGURE. S25. MS spectrum of herniarin.

SUPPLEMENTARY FIGURE. S26. MS spectrum of hederagenin.

SUPPLEMENTARY FIGURE. S27. MS spectrum of oleanolic acid.

SUPPLEMENTARY FIGURE. S28. MS spectrum of β -Sitosterol.

SUPPLEMENTARY FIGURE. S29. MS spectrum of maslinic acid.

SUPPLEMENTARY FIGURE. S30. MS spectrum of lupeol.

SUPPLEMENTARY FIGURE. S31. MS spectrum of betulonic acid.

SUPPLEMENTARY FIGURE. S32. Depiction of kaempferol library record in Bruker Library Editor 4.4.

SUPPLEMENTARY FIGURE. S33. Depiction of the common fragmentation pathway of the investigated analyte.

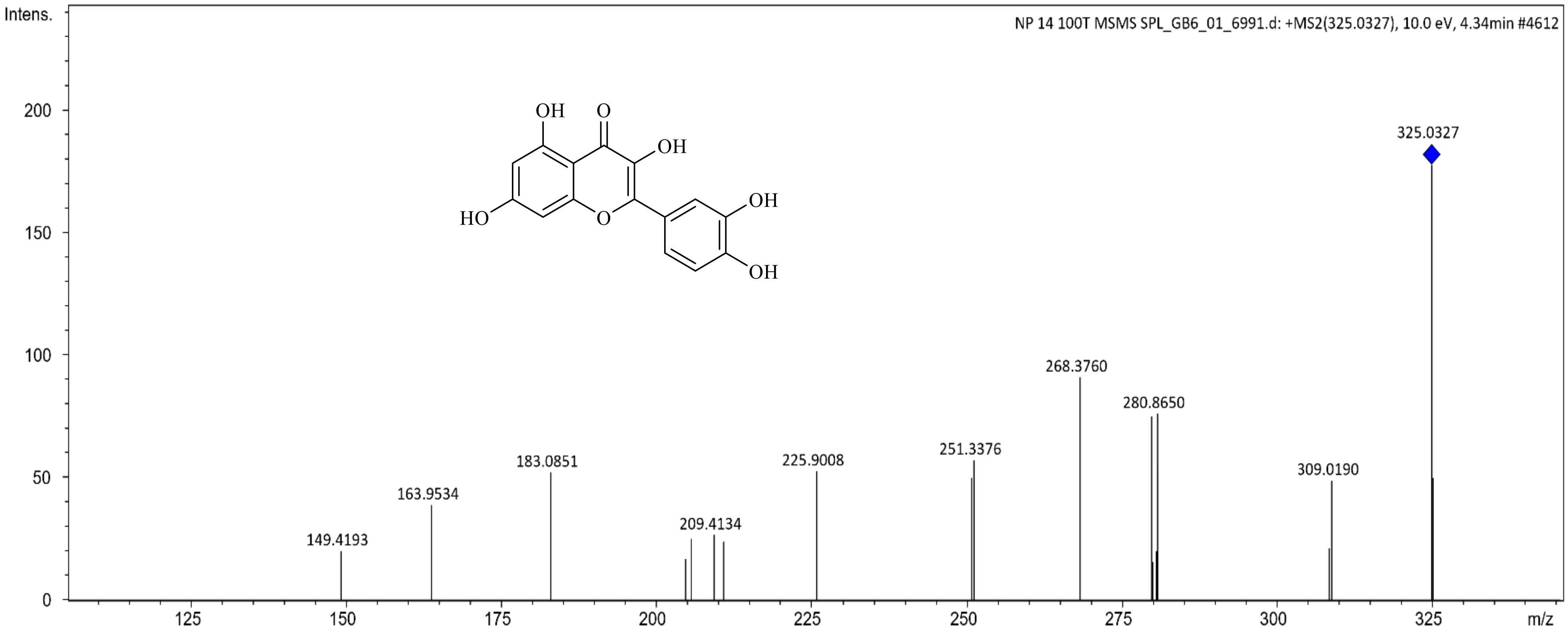

SUPPLEMENTARY FIGURE. S1. MS spectrum of quercetin.

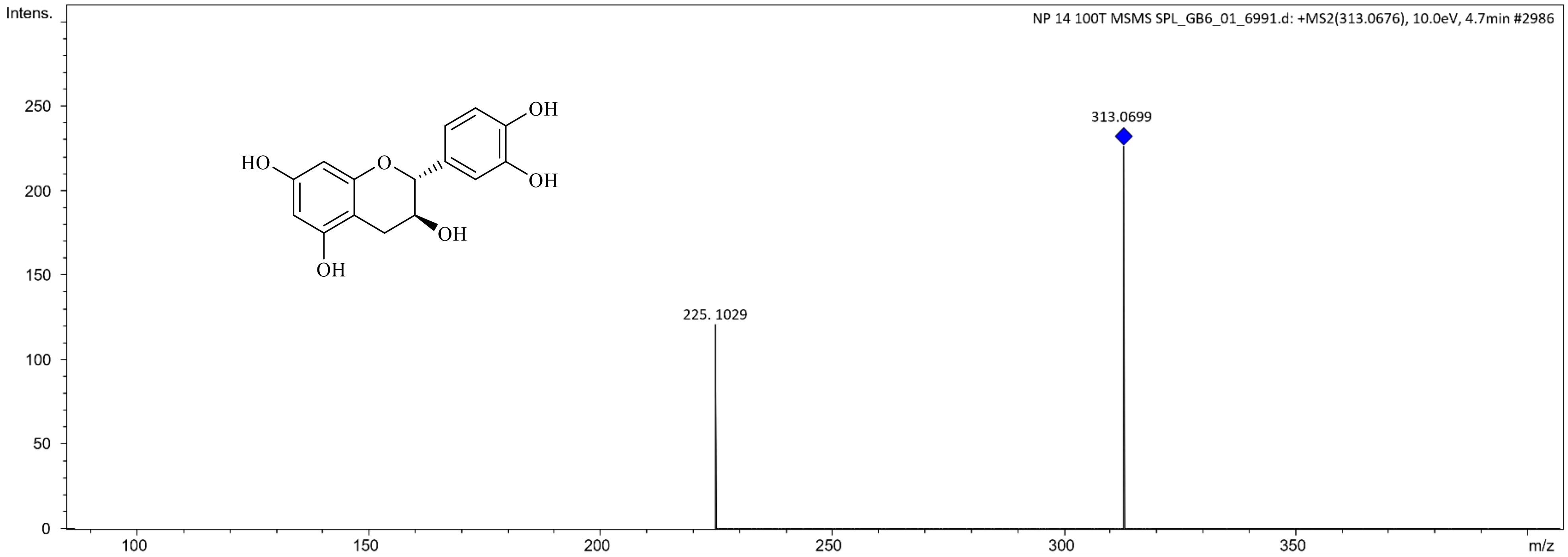

SUPPLEMENTARY FIGURE. S2. MS spectrum of catechin.

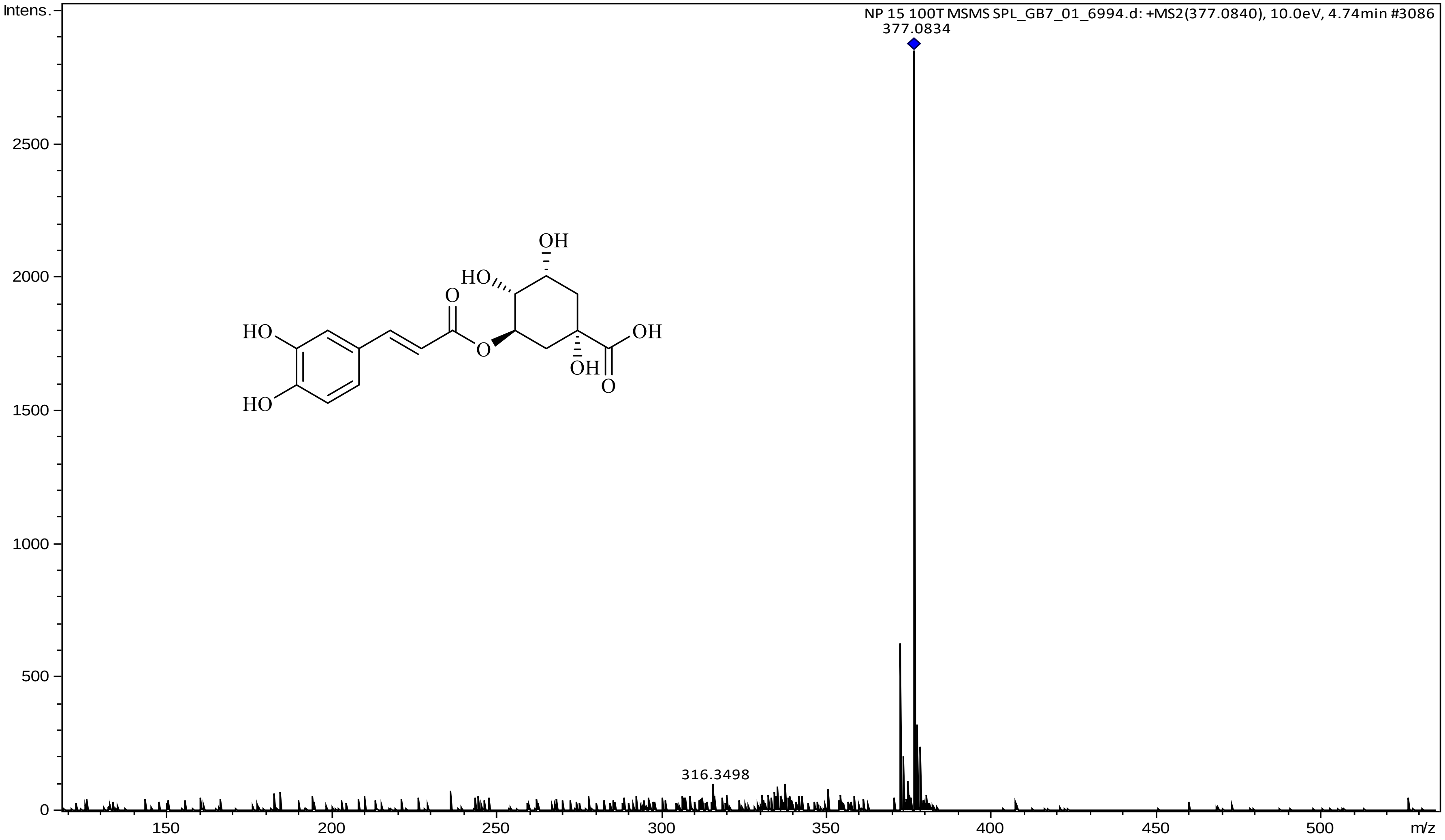

SUPPLEMENTARY FIGURE. S3 MS spectrum of chlorogenic acid.

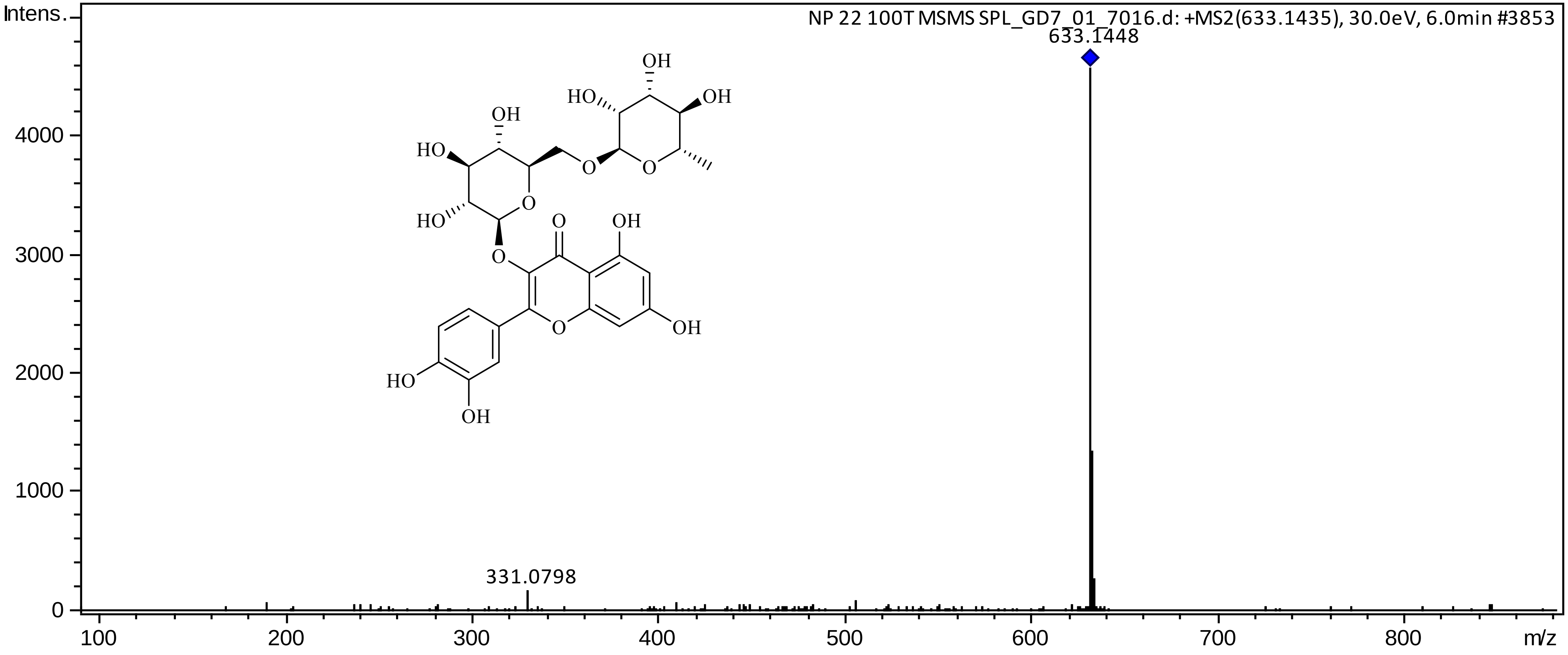

SUPPLEMENTARY FIGURE. S4. MS spectrum of rutin.

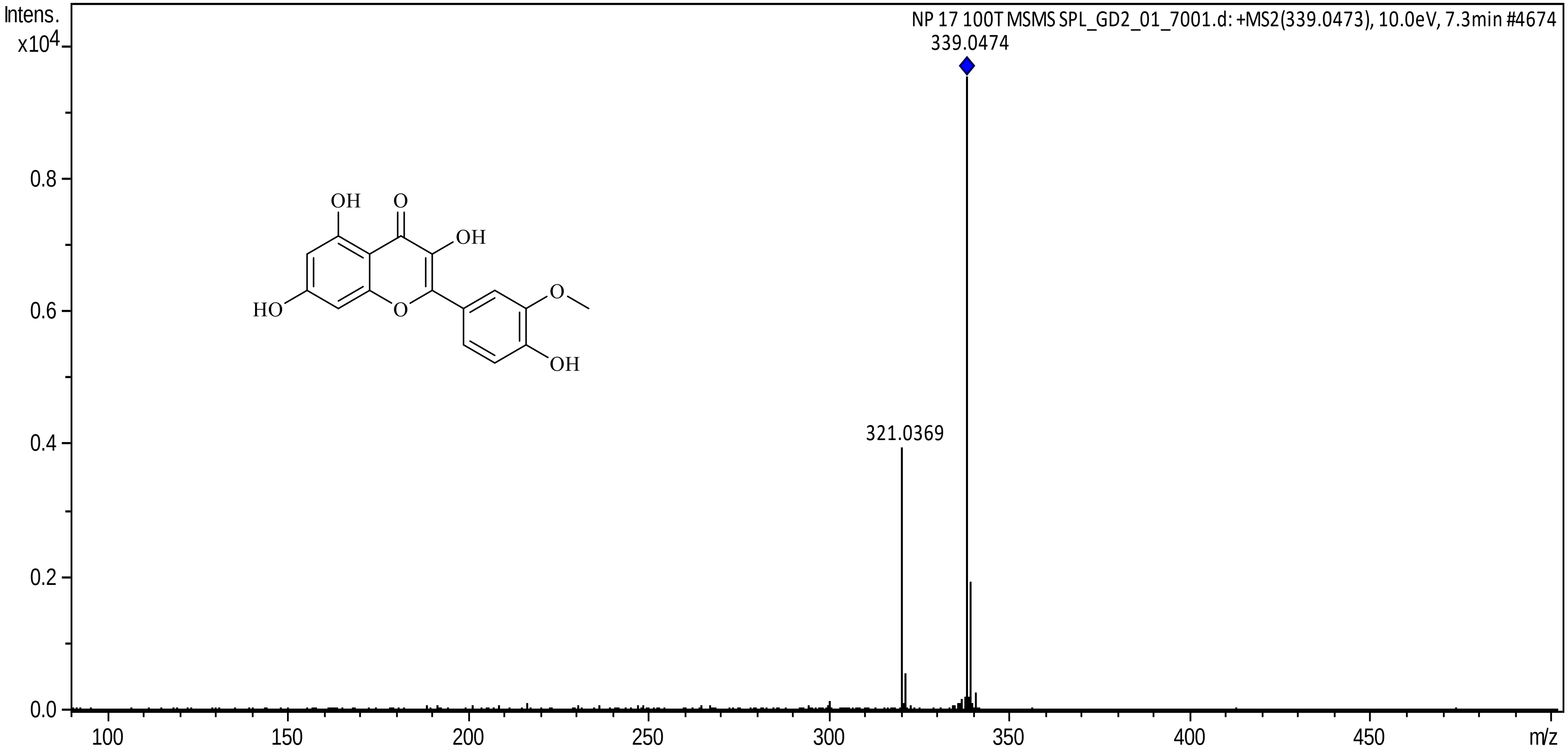

SUPPLEMENTARY FIGURE. S5. MS spectrum of isorhamnetin.

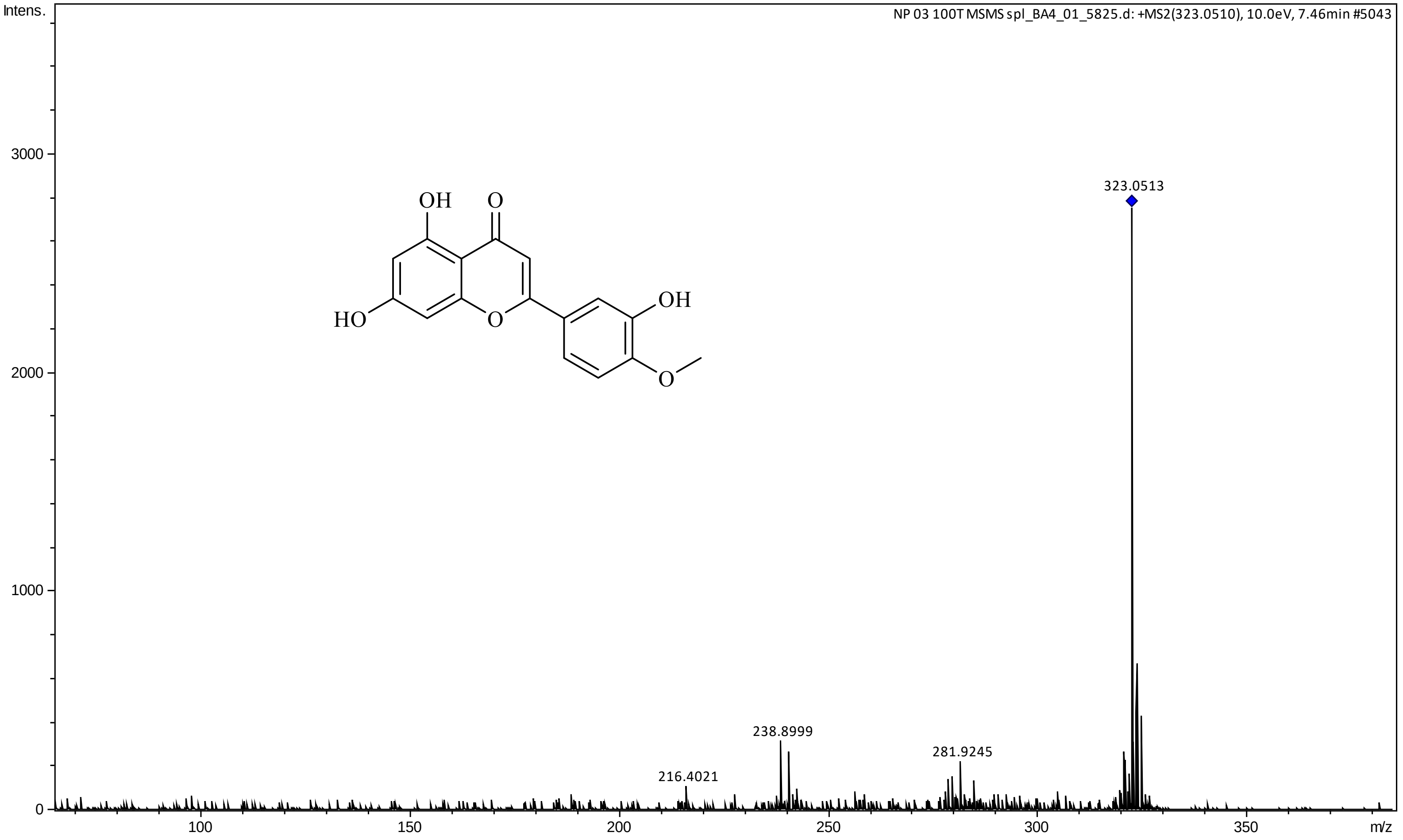

SUPPLEMENTARY FIGURE. S6. MS spectrum of diosmetin.

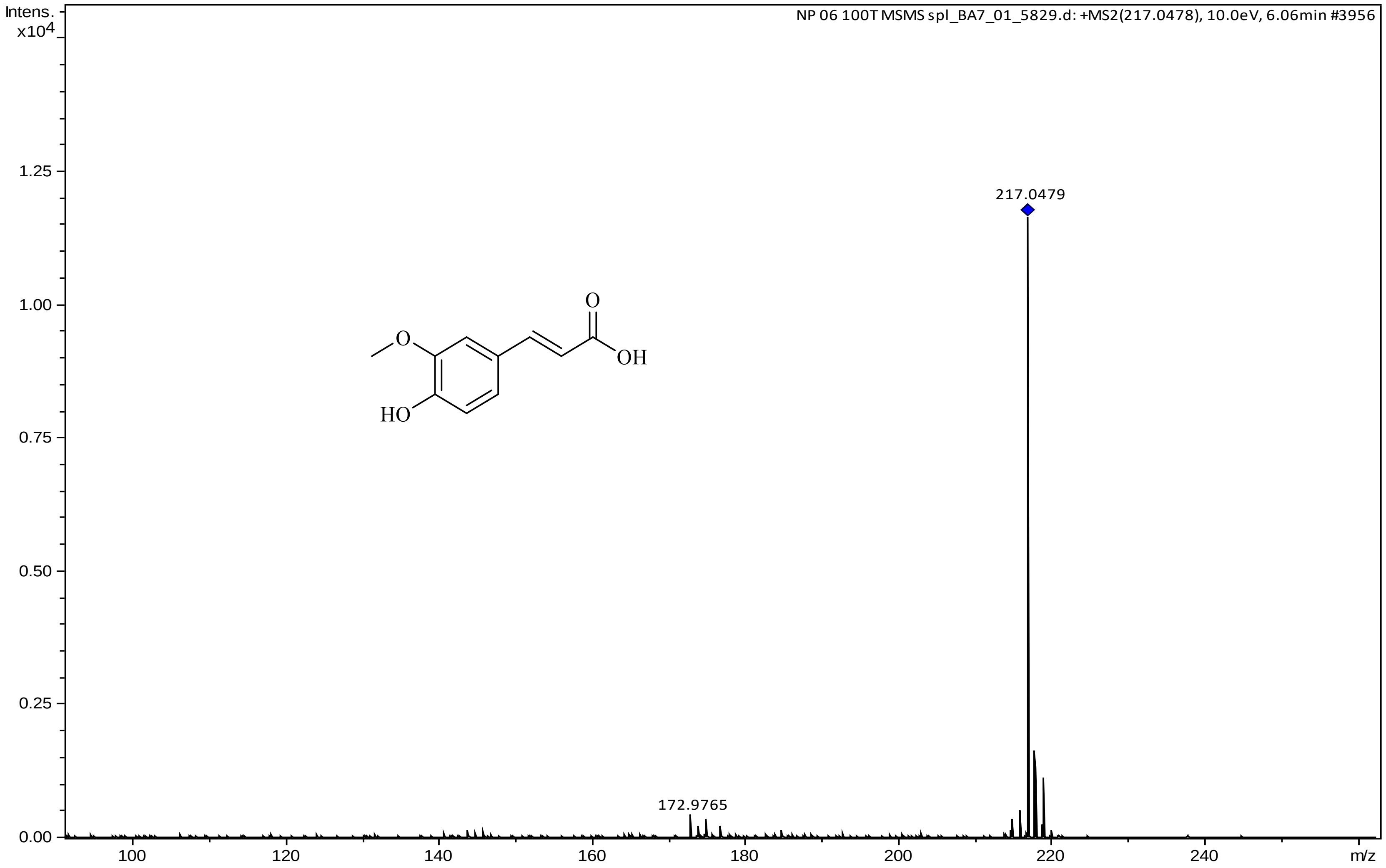

SUPPLEMENTARY FIGURE. S7. MS spectrum of *trans*-ferulic acid.

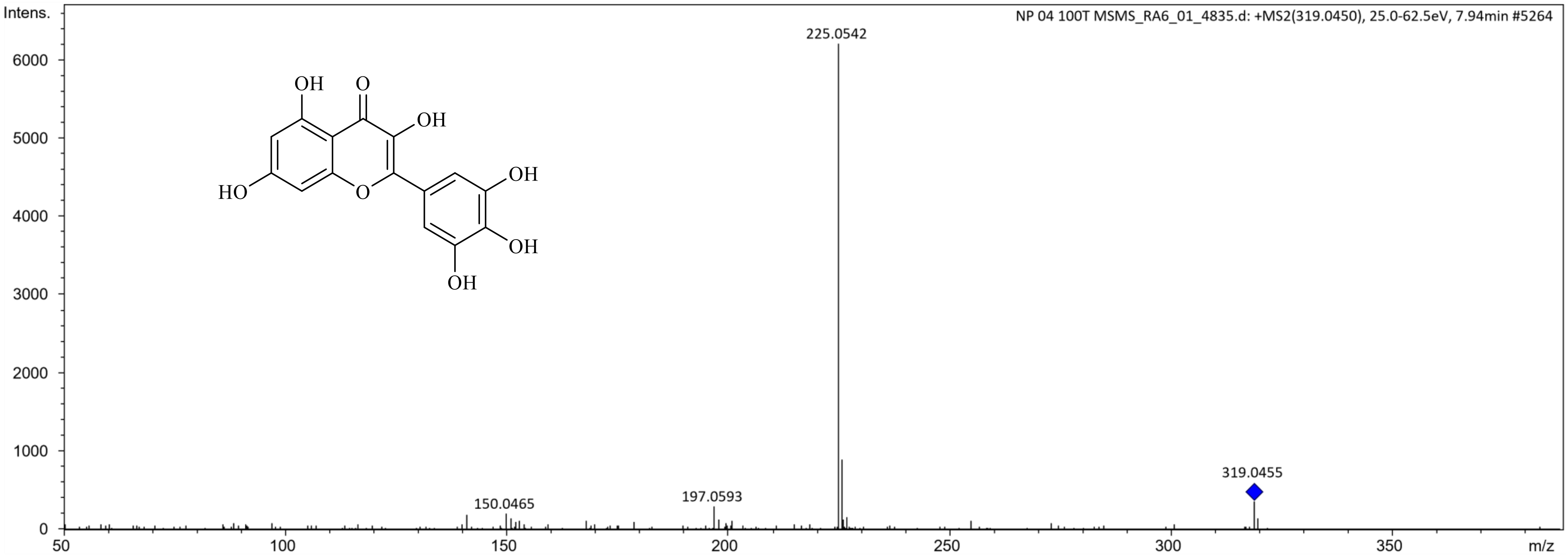

SUPPLEMENTARY FIGURE. S8. MS spectrum of myricetin.

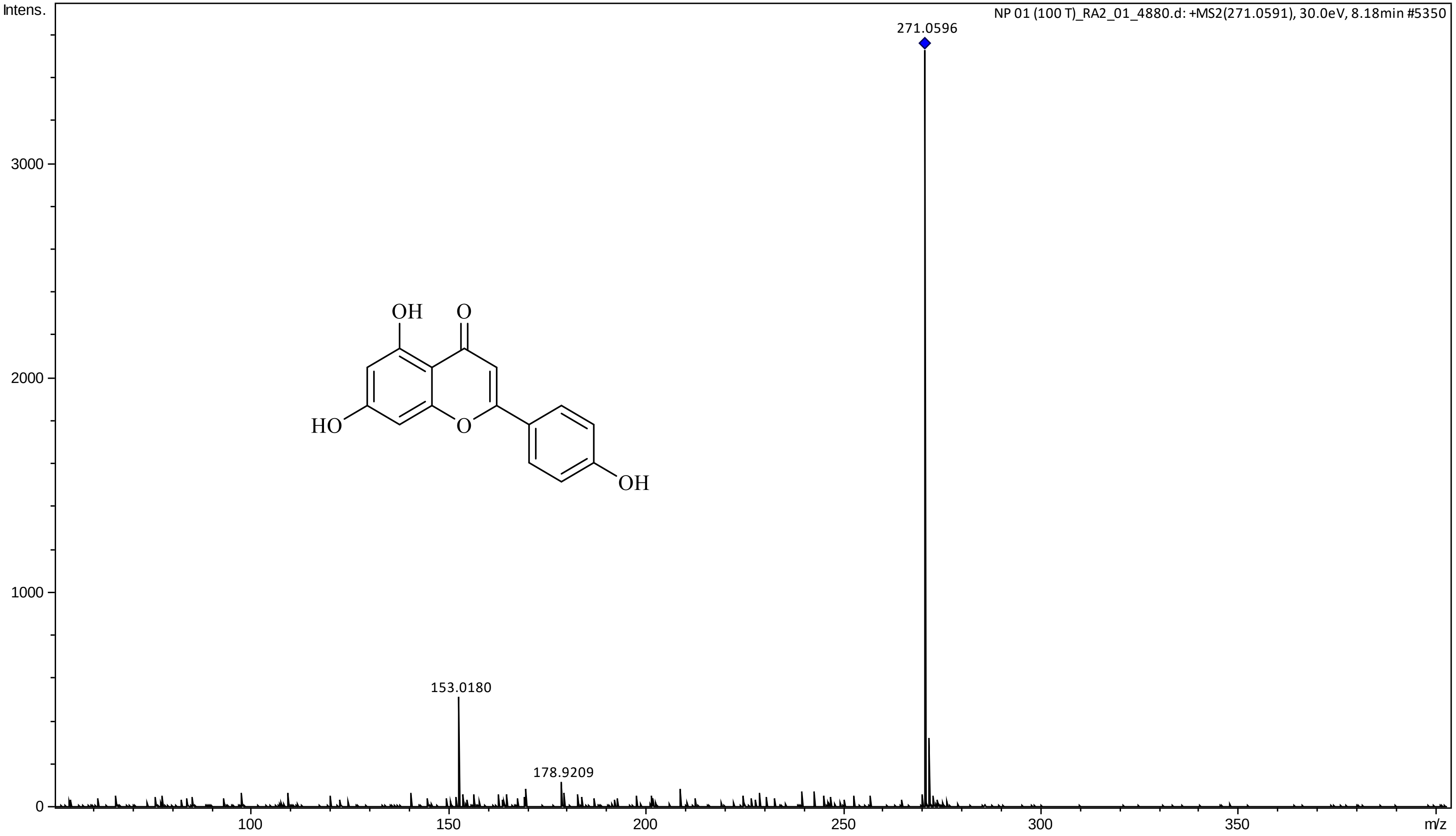

SUPPLEMENTARY FIGURE. S9. MS spectrum of apigenin.

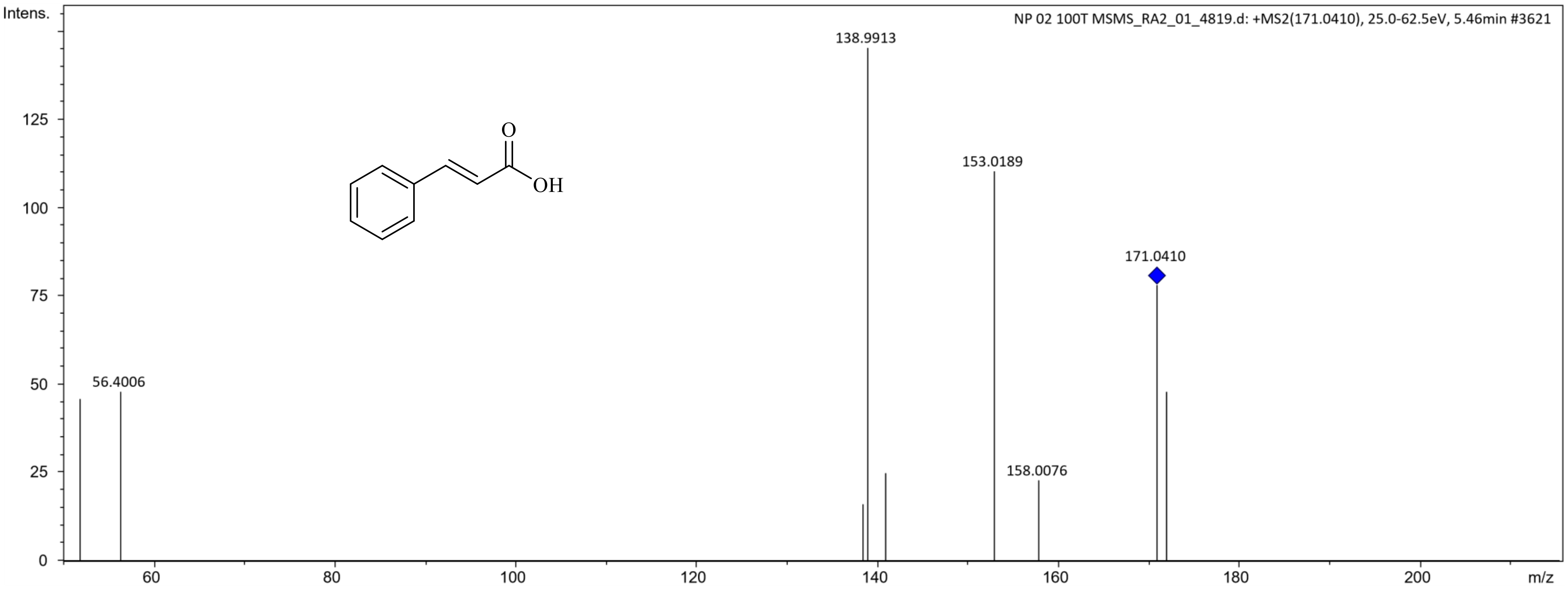

SUPPLEMENTARY FIGURE. S10. MS spectrum of cinnamic acid.

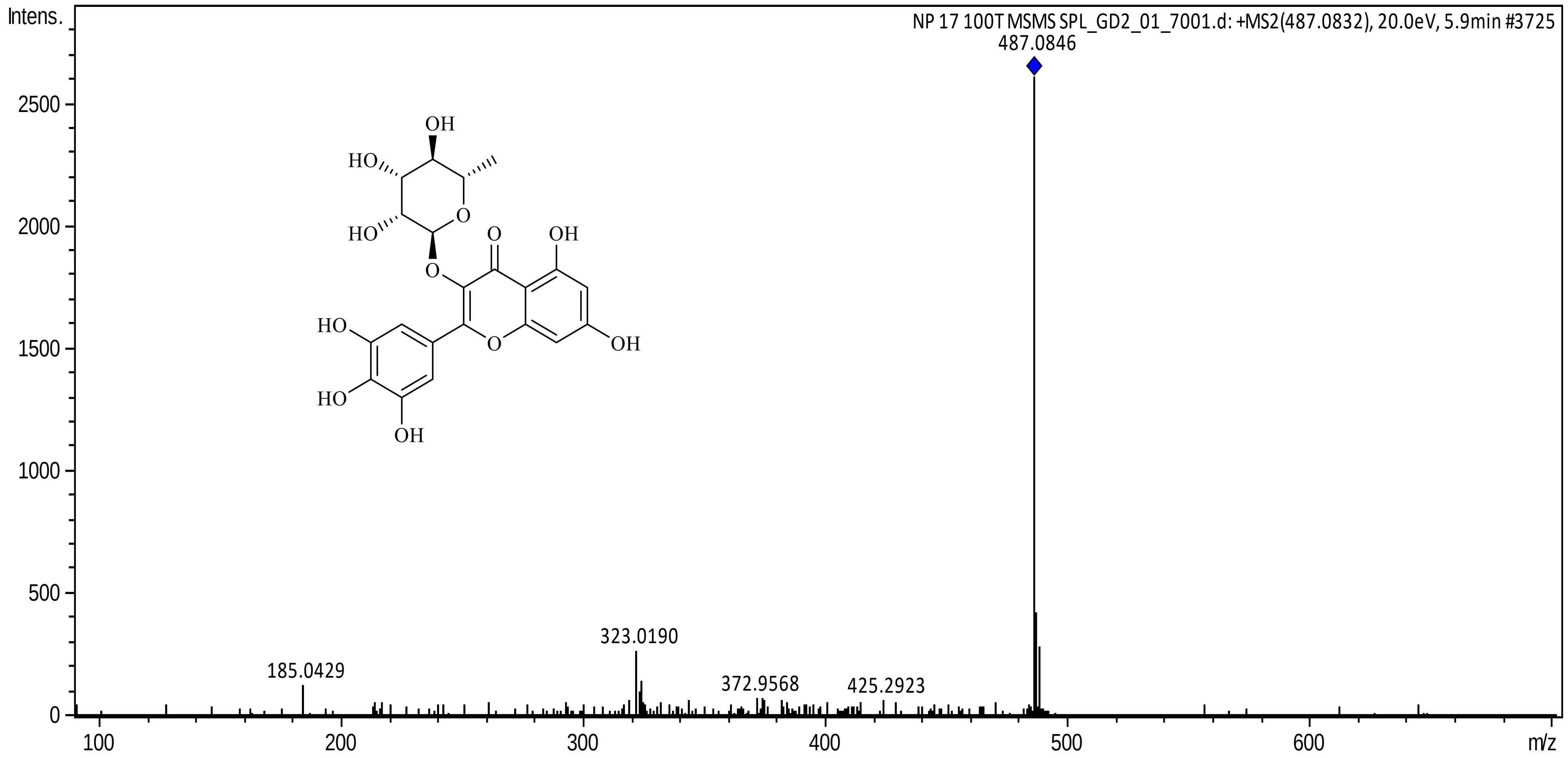

SUPPLEMENTARY FIGURE. S11. MS spectrum of myricitrin.

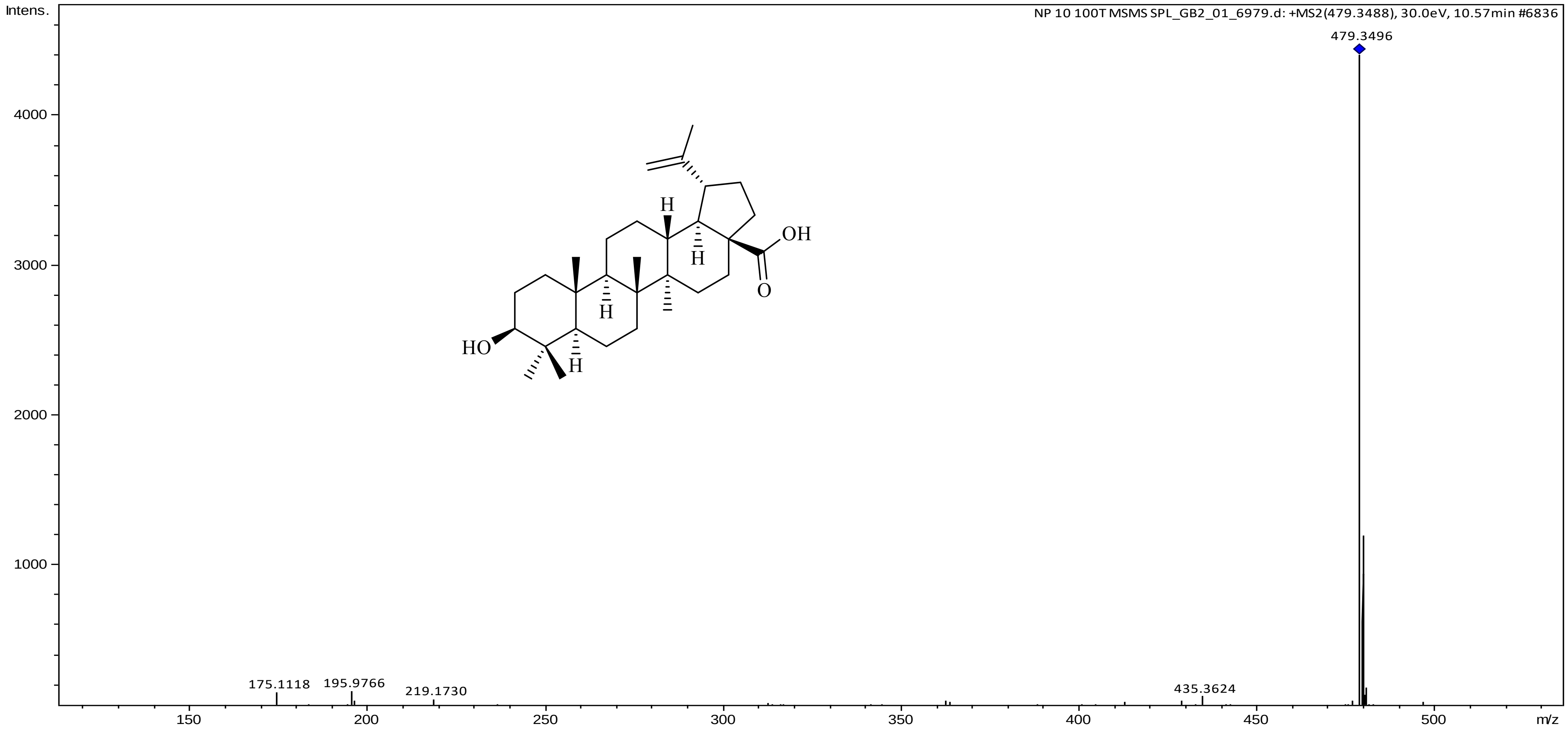

SUPPLEMENTARY FIGURE. S12. MS spectrum of betulinic acid.

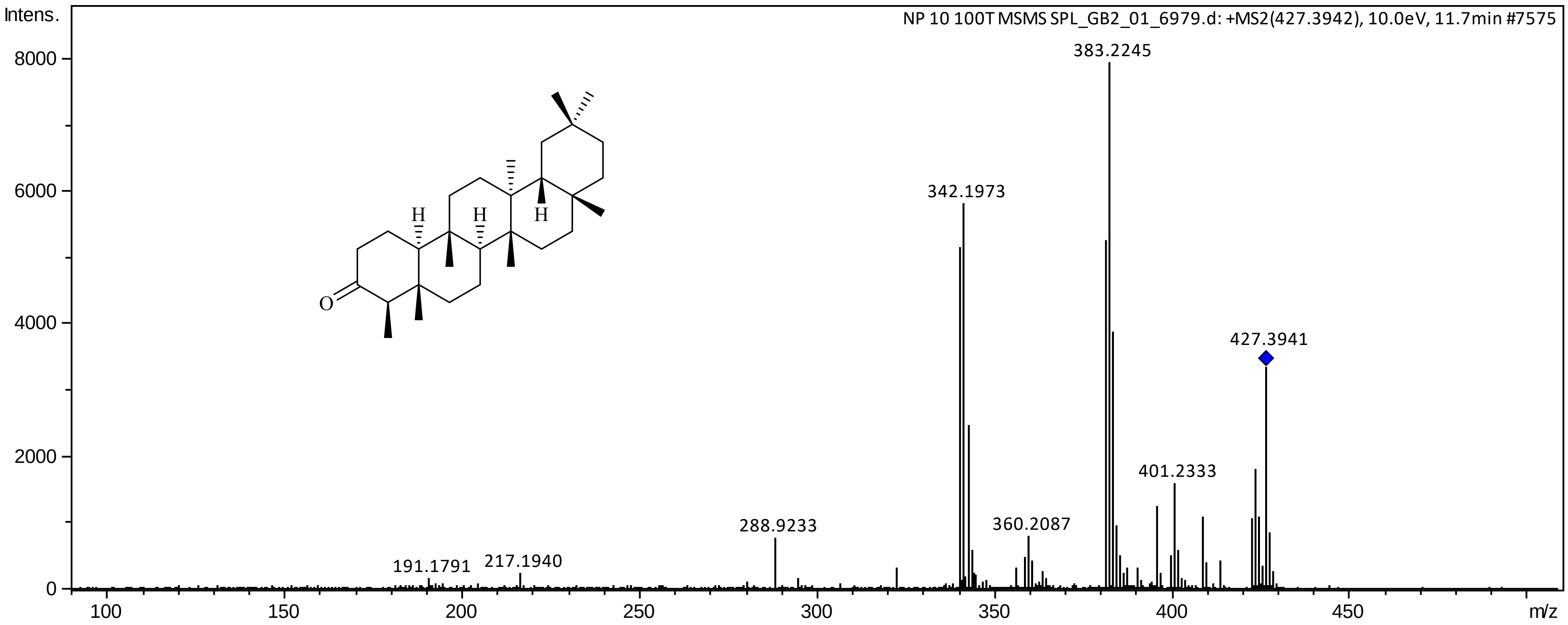

SUPPLEMENTARY FIGURE. S13. MS spectrum of friedelin.

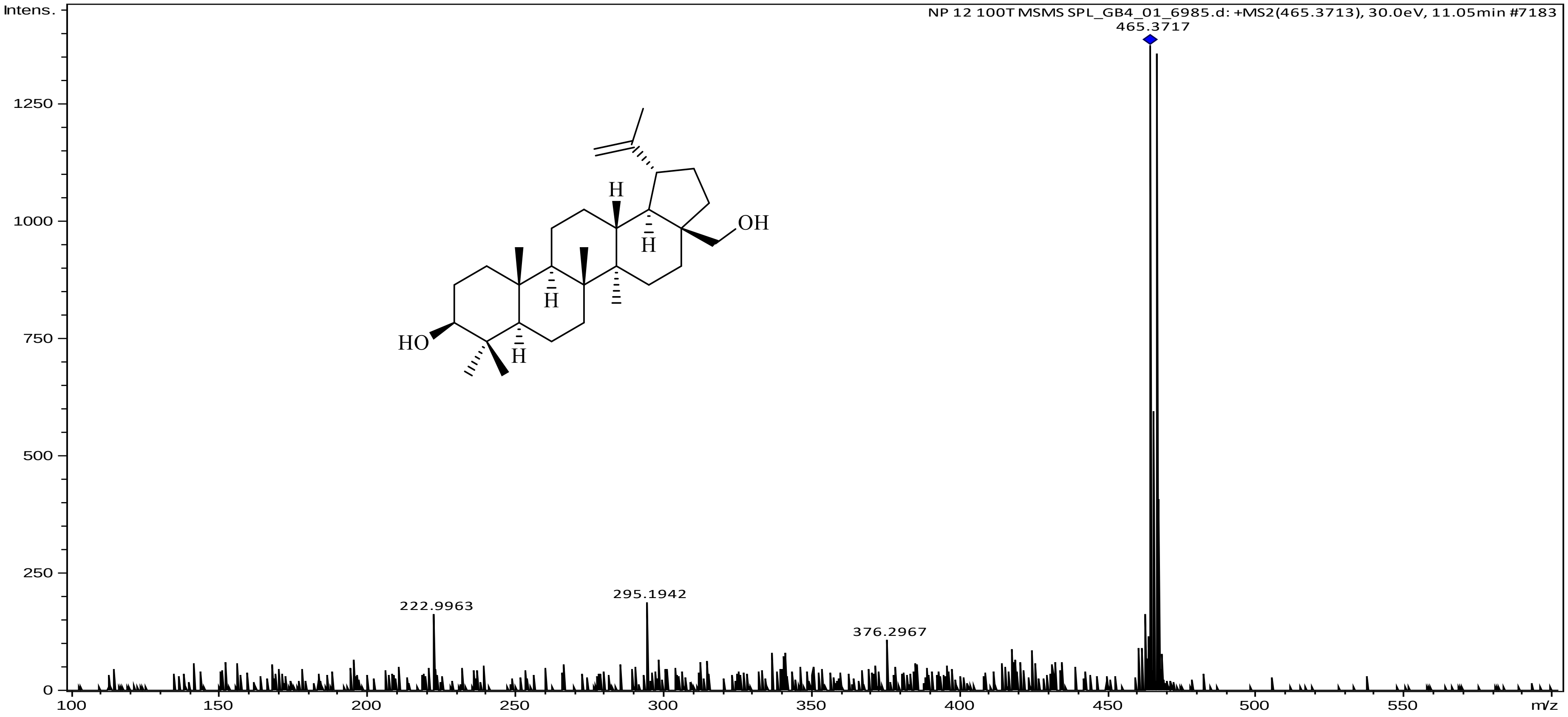

SUPPLEMENTARY FIGURE. S14. MS spectrum of betulin.

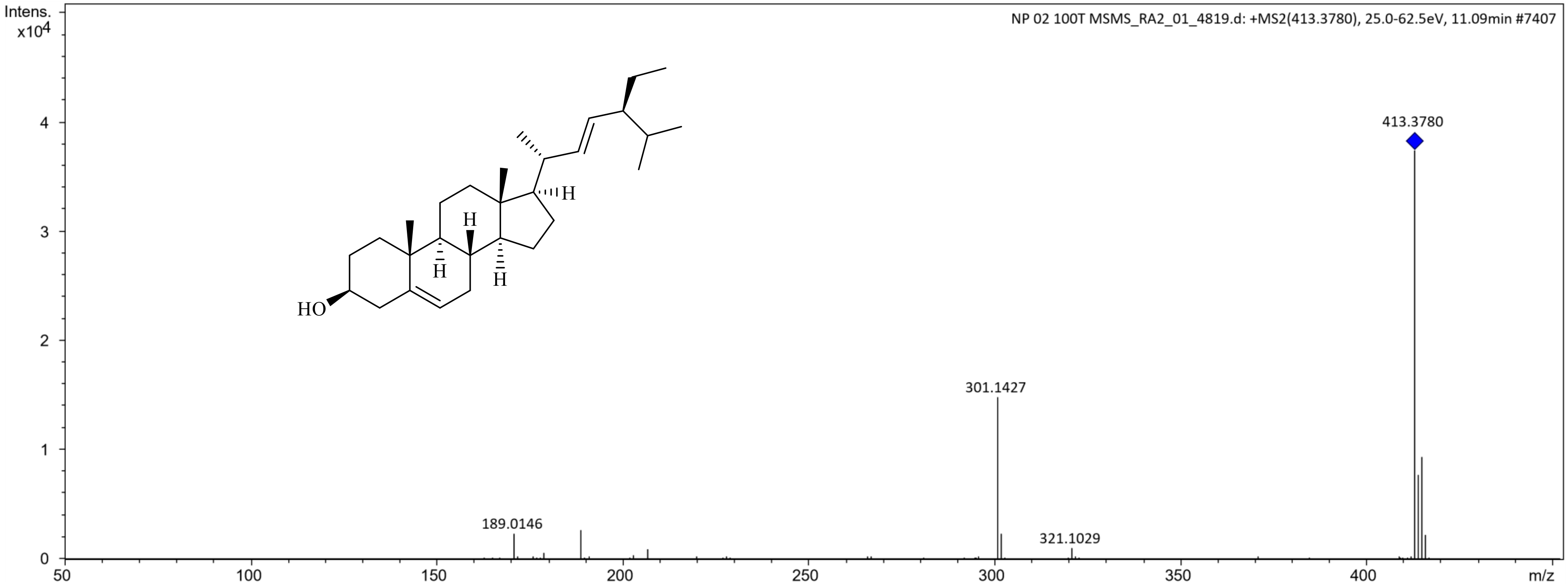

SUPPLEMENTARY FIGURE. S15. MS spectrum of stigmasterol.

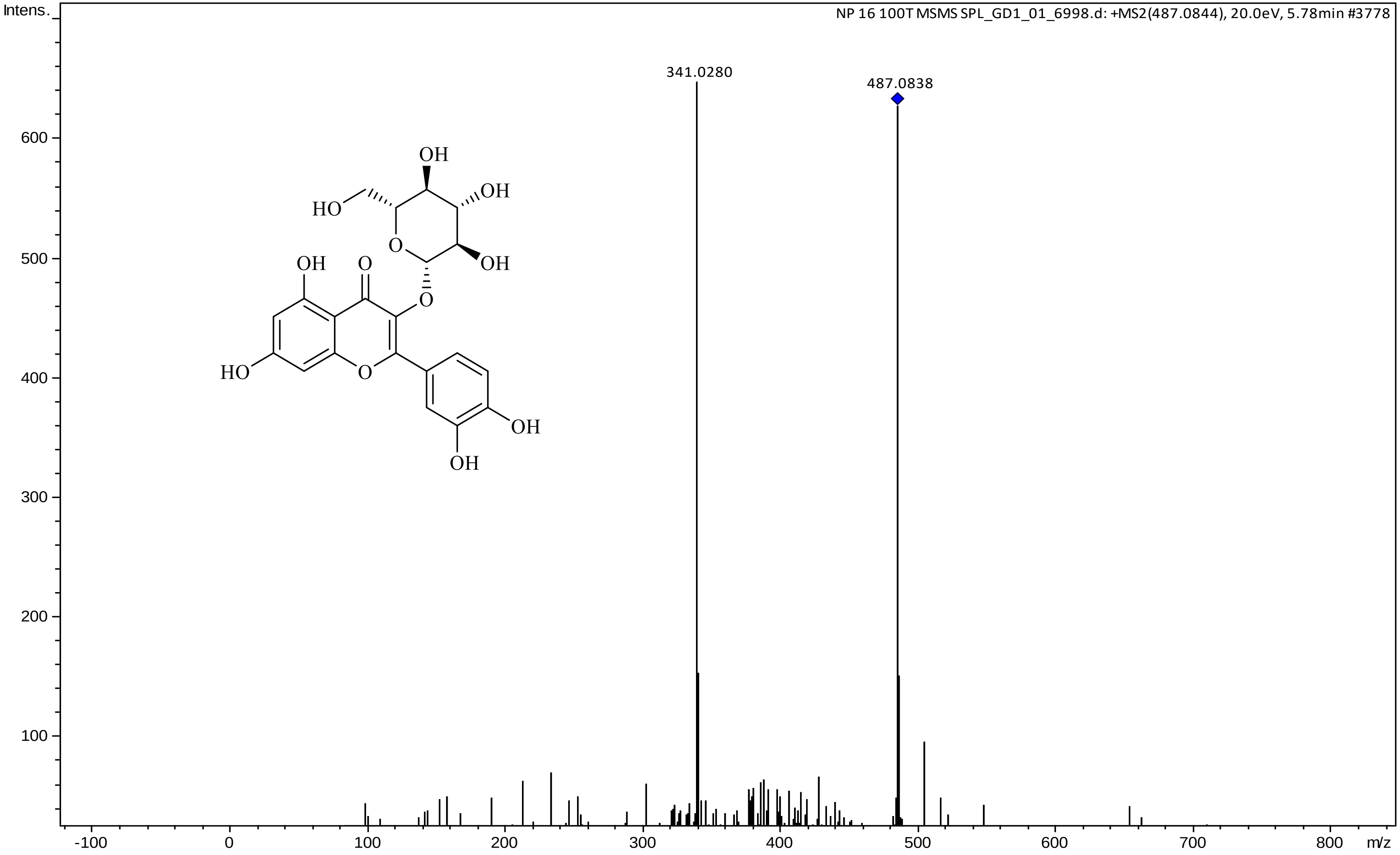

SUPPLEMENTARY FIGURE. S16. MS spectrum of isoquercitrin.

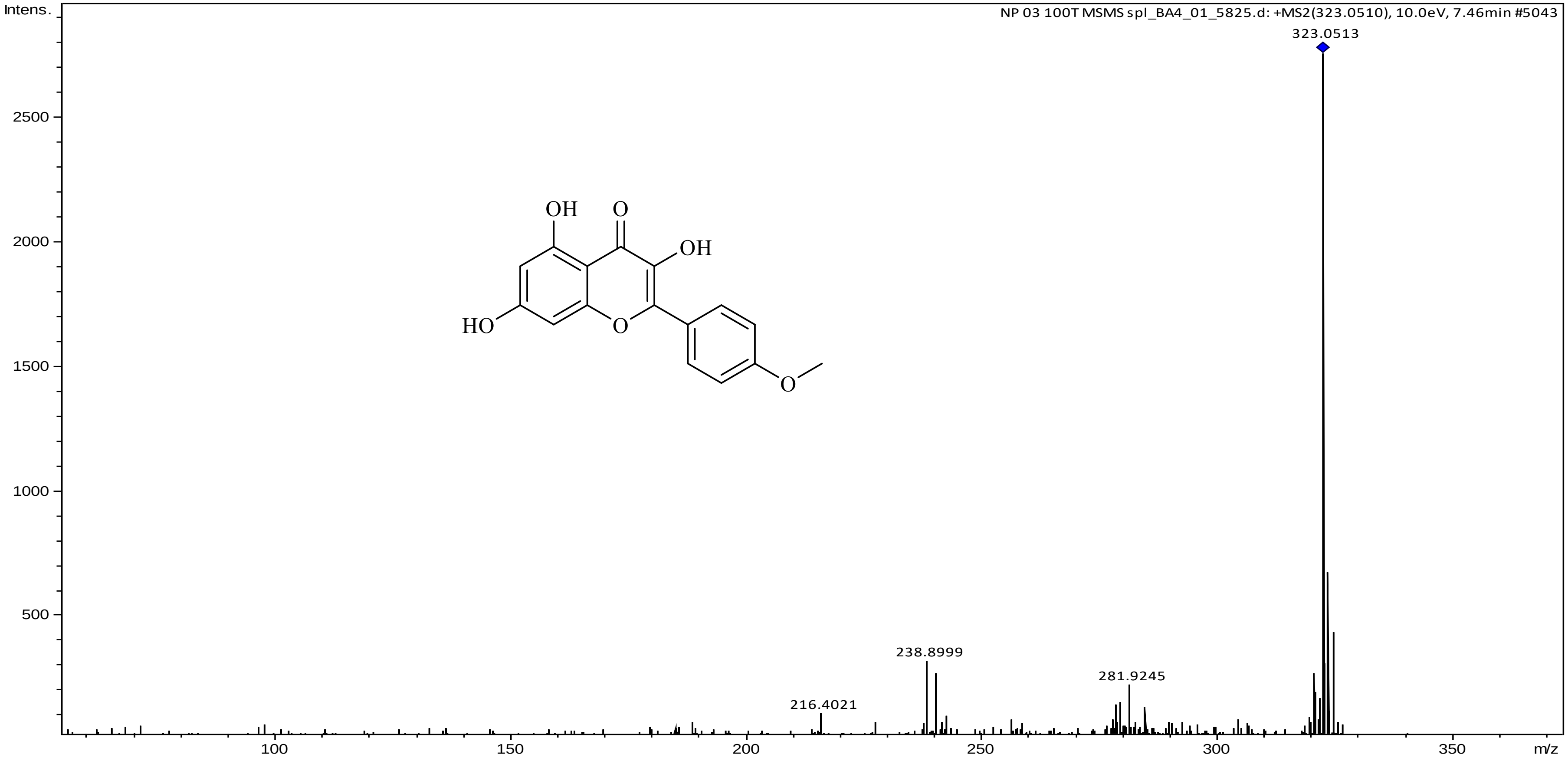

SUPPLEMENTARY FIGURE. S17. MS spectrum of kaempferide (kaempferol 4'-O-methyl ether).

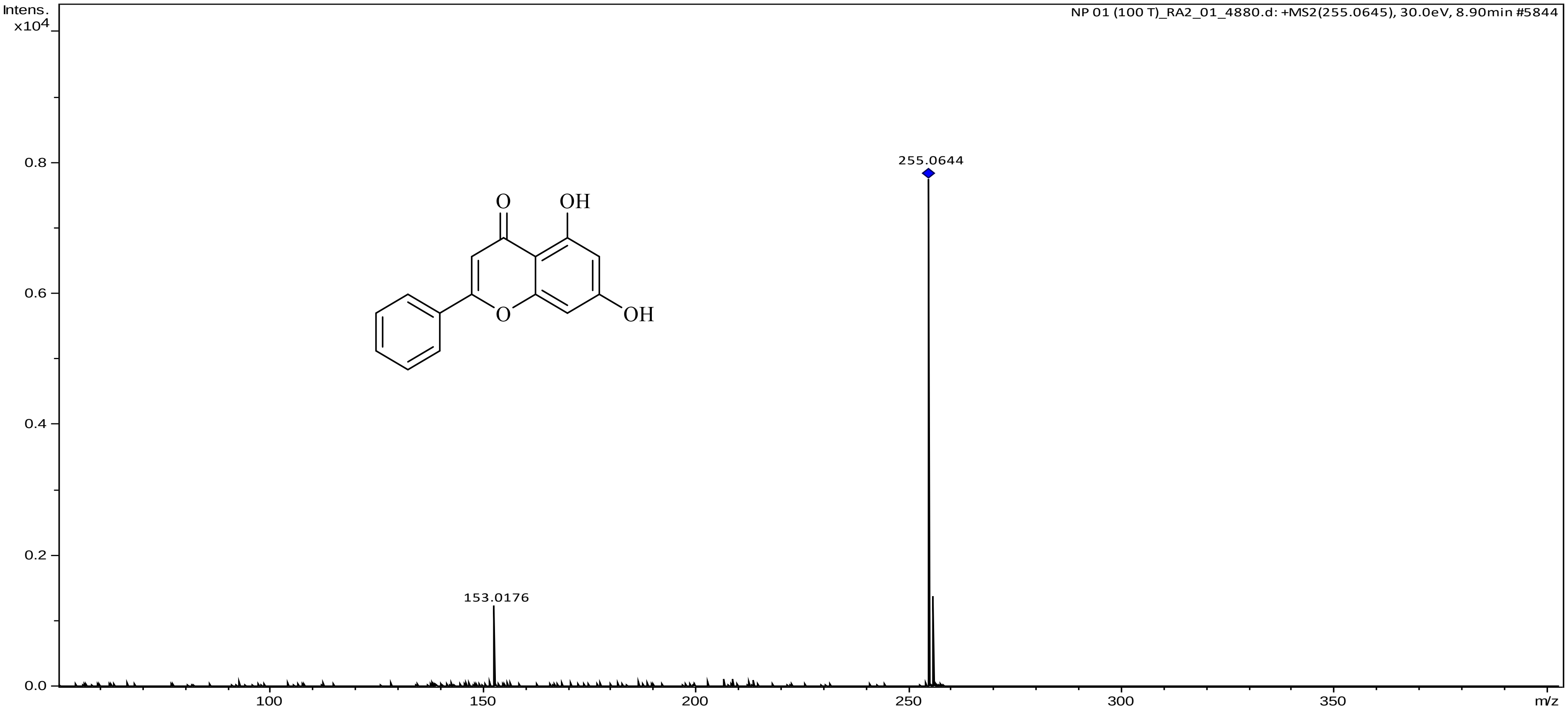

SUPPLEMENTARY FIGURE. S18. MS spectrum of chrysin.

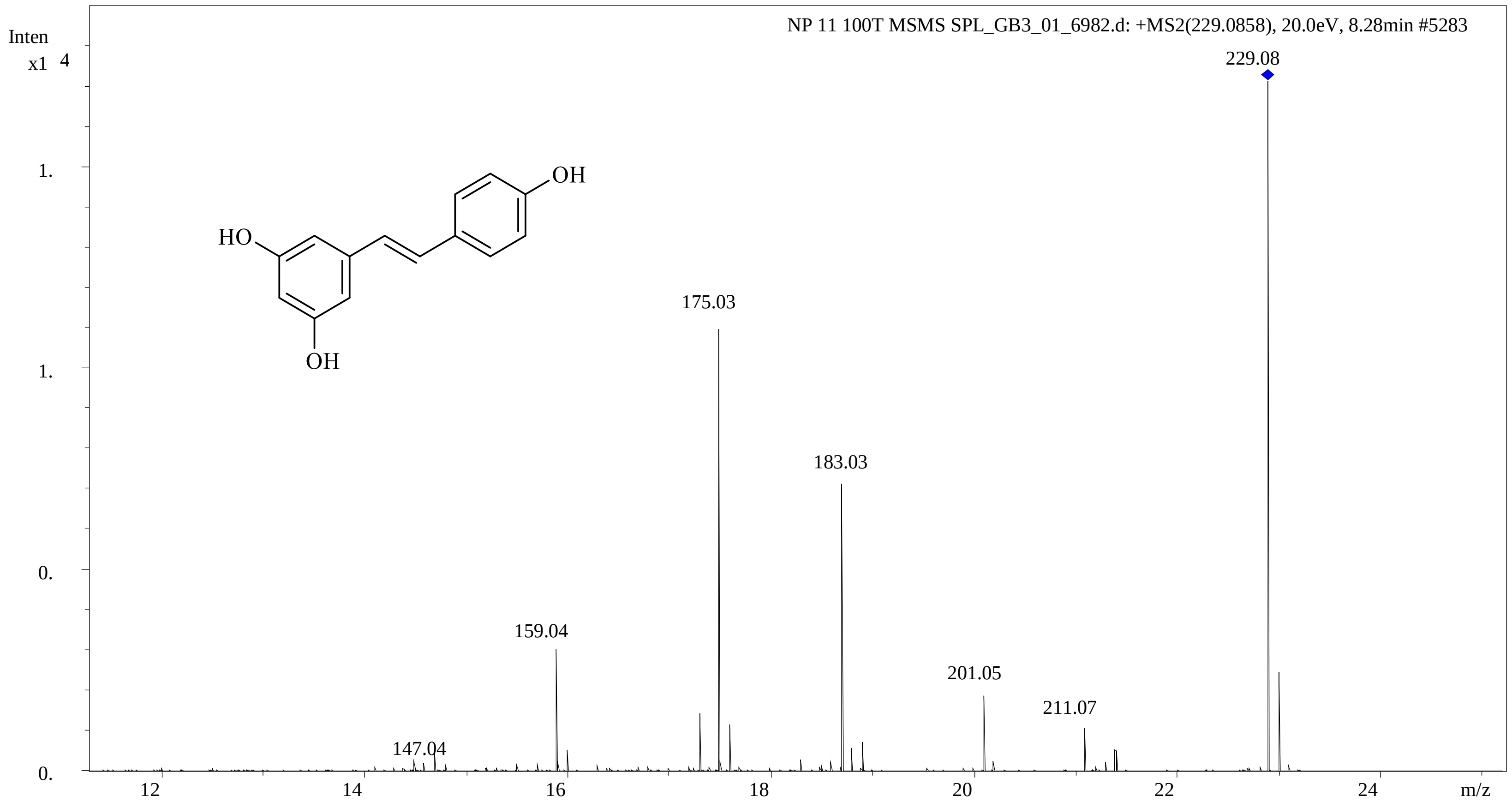

SUPPLEMENTARY FIGURE. S19. MS spectrum of *trans*-resveratrol.

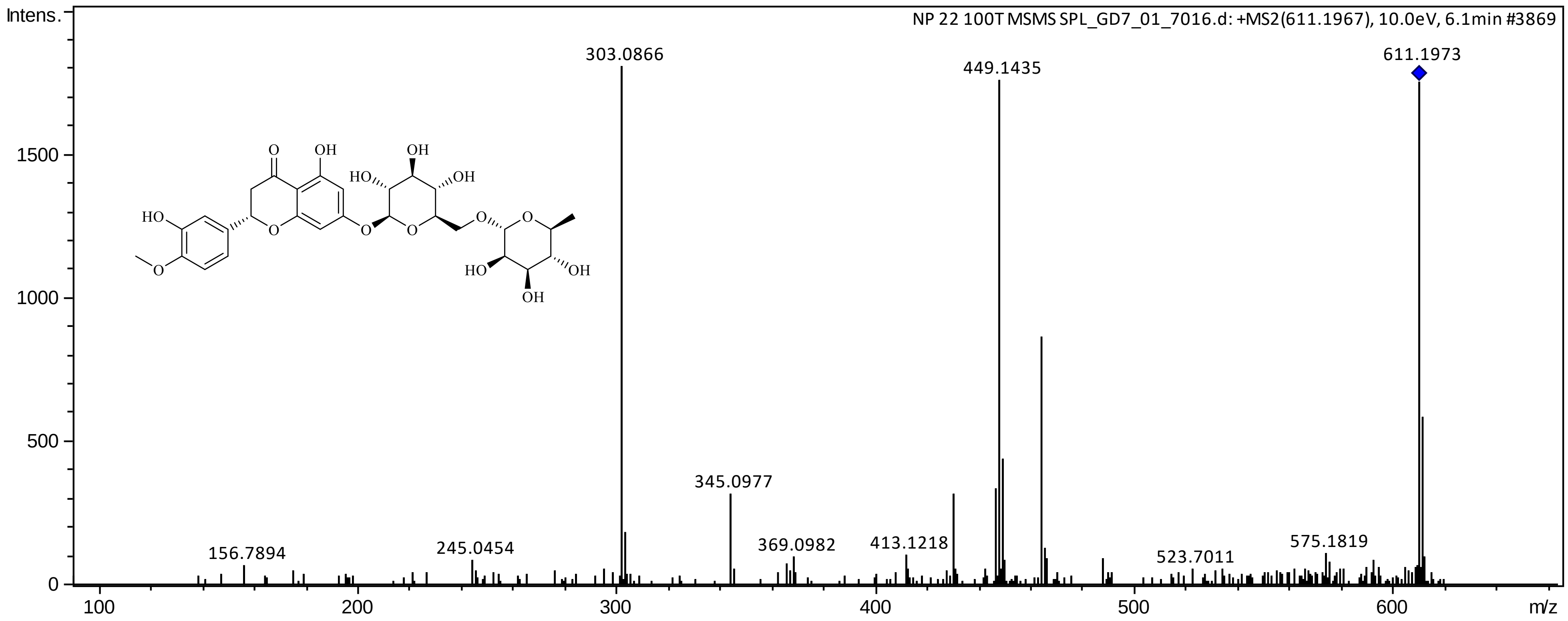

SUPPLEMENTARY FIGURE. S20. MS spectrum of hesperidin.

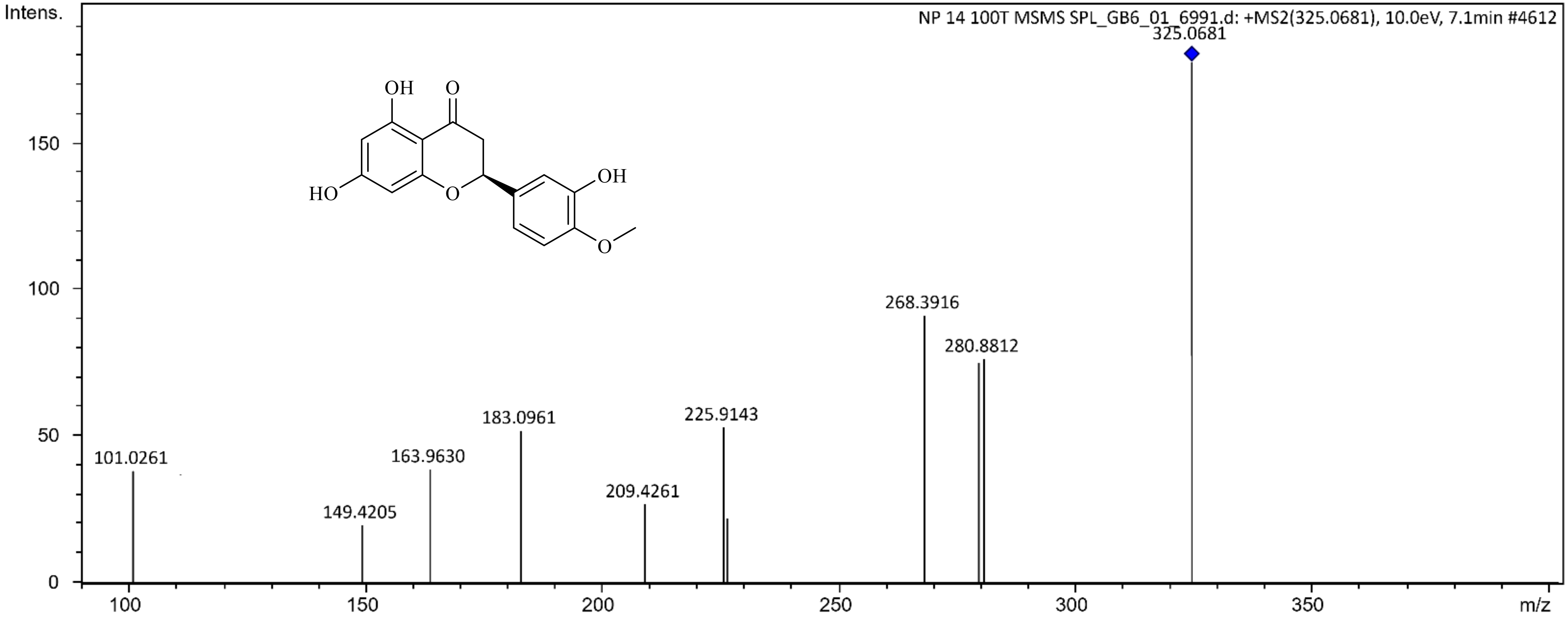

SUPPLEMENTARY FIGURE. S21. MS spectrum of hesperetin.

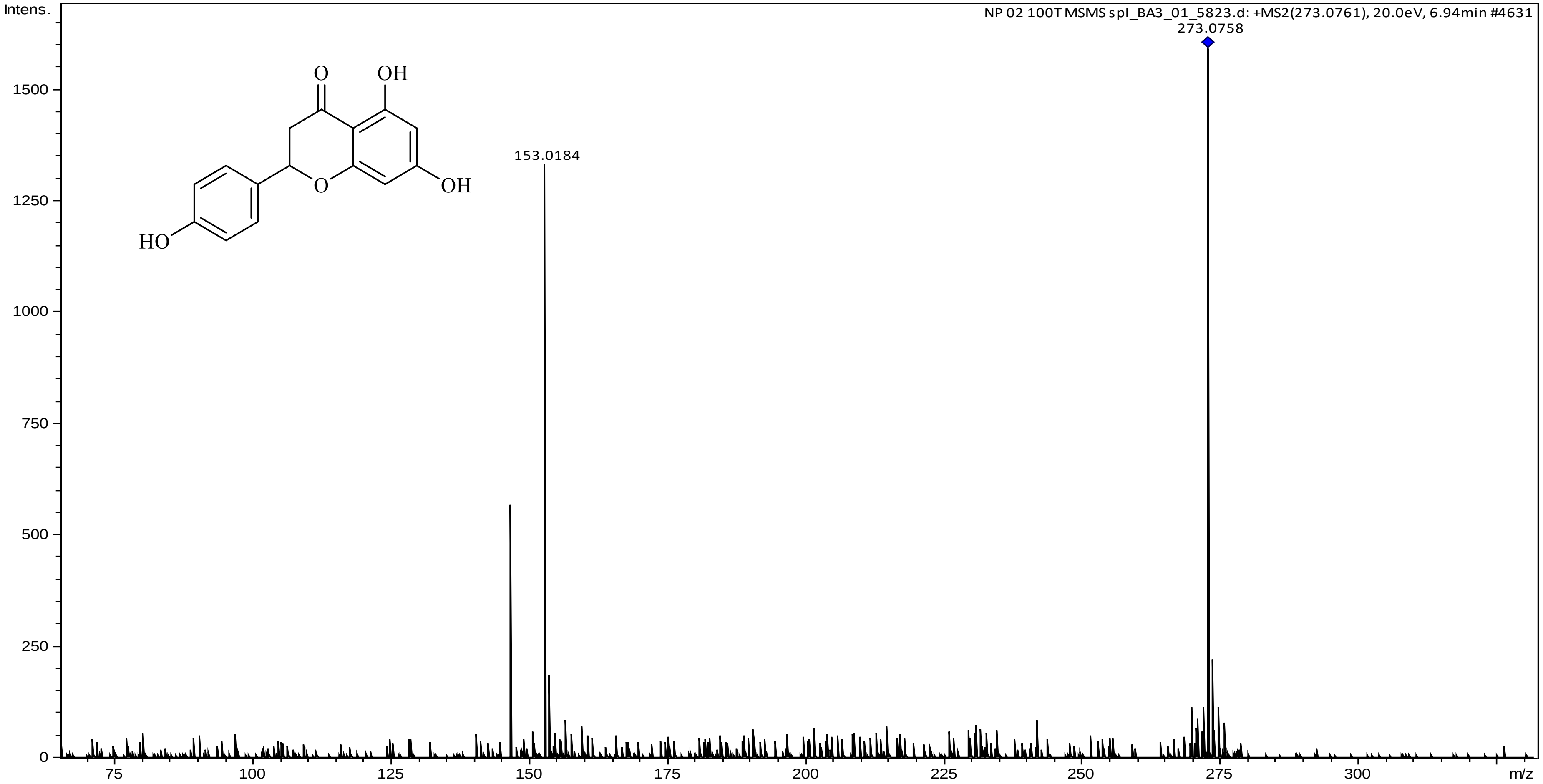

SUPPLEMENTARY FIGURE. S22. MS spectrum of naringenin.

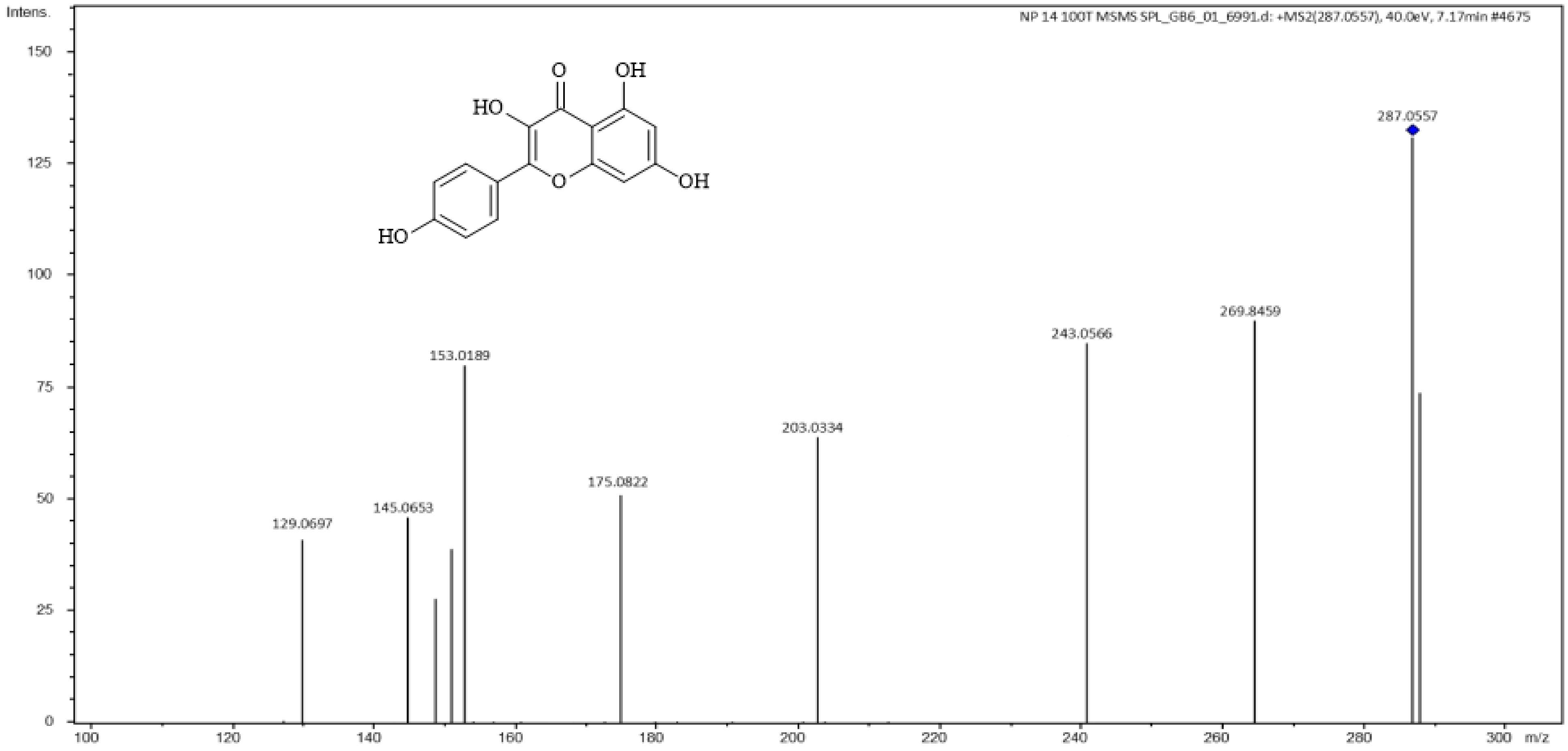

SUPPLEMENTARY FIGURE. S23. MS spectrum of kaempferol.

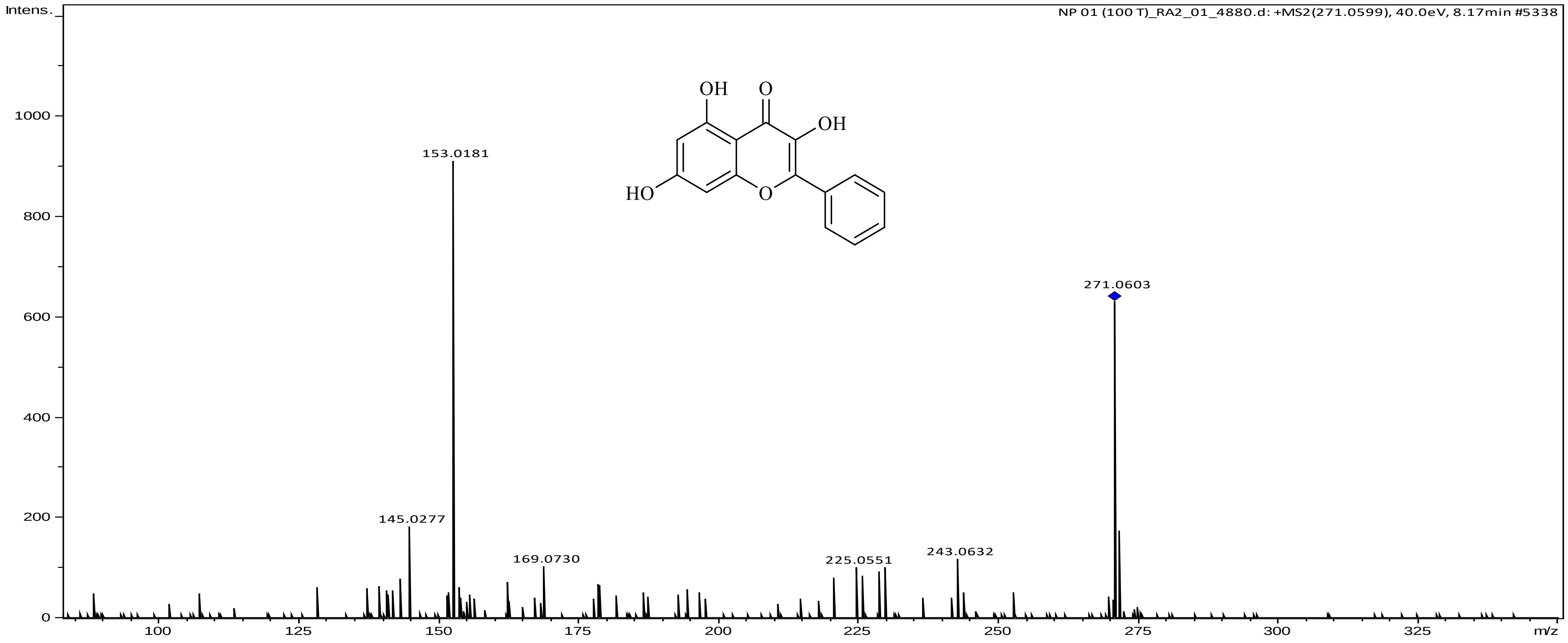

SUPPLEMENTARY FIGURE. S24. MS spectrum of galangin.

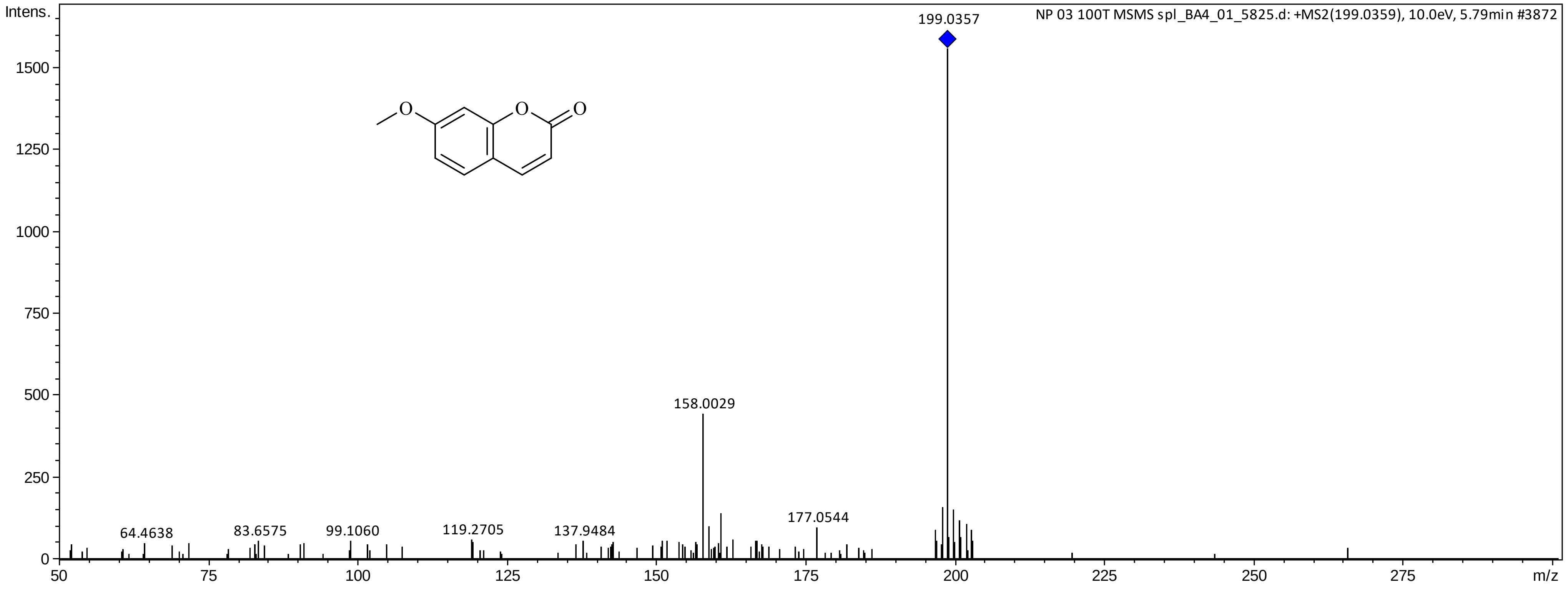

SUPPLEMENTARY FIGURE. S25. MS spectrum of herniarin.

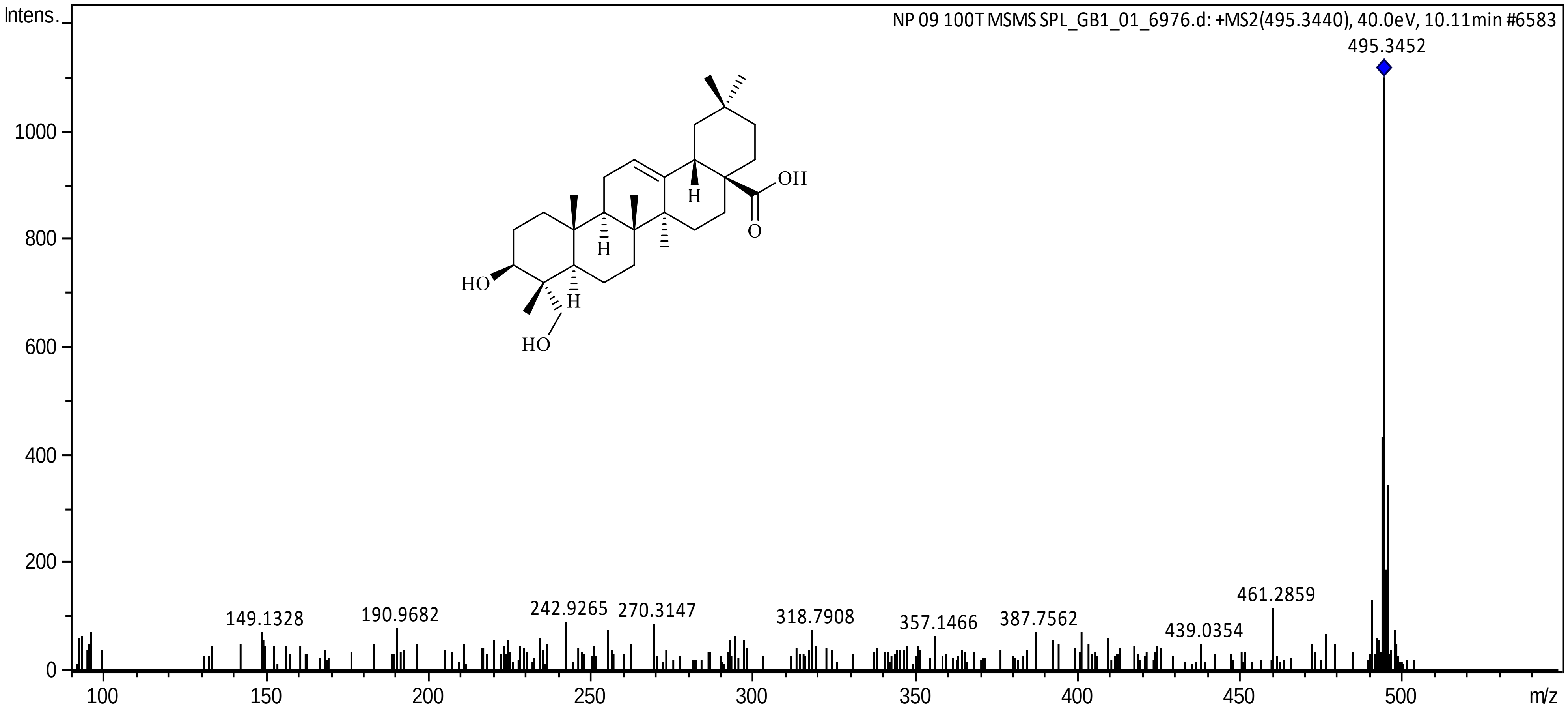

SUPPLEMENTARY FIGURE. S26. MS spectrum of hederagenin.

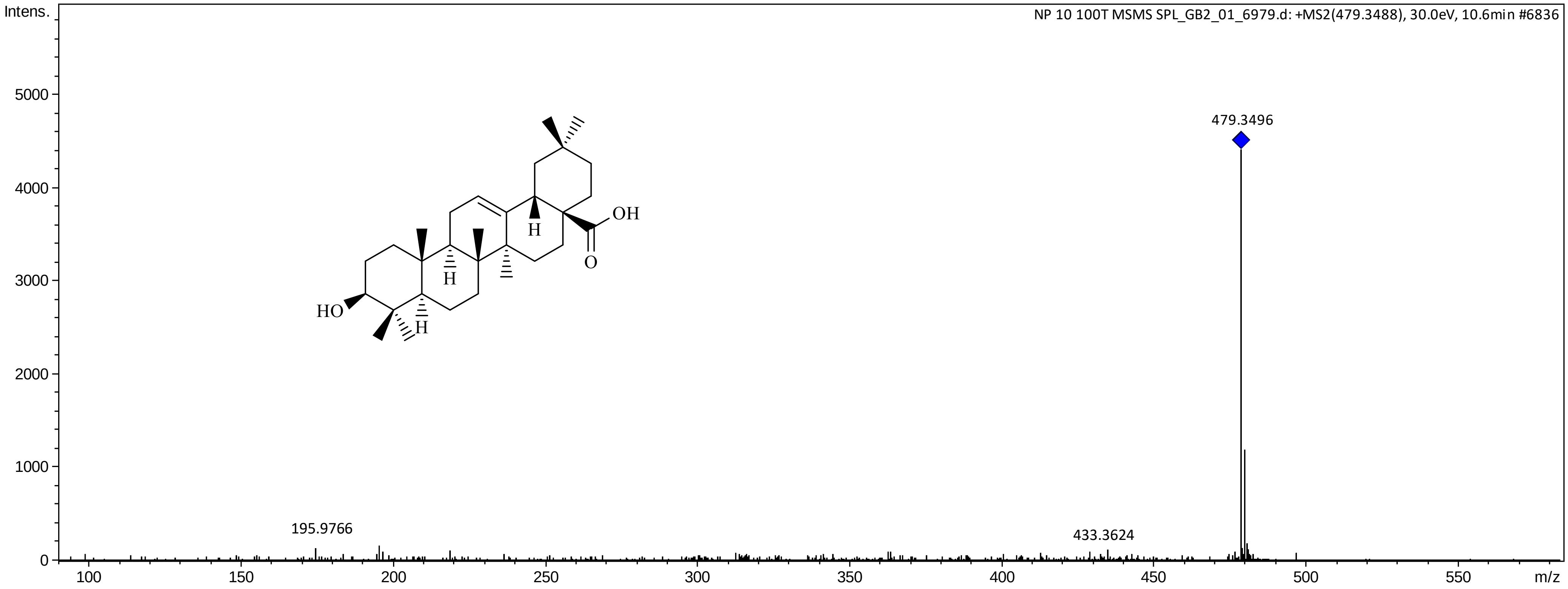

SUPPLEMENTARY FIGURE. S27. MS spectrum of oleanolic acid.

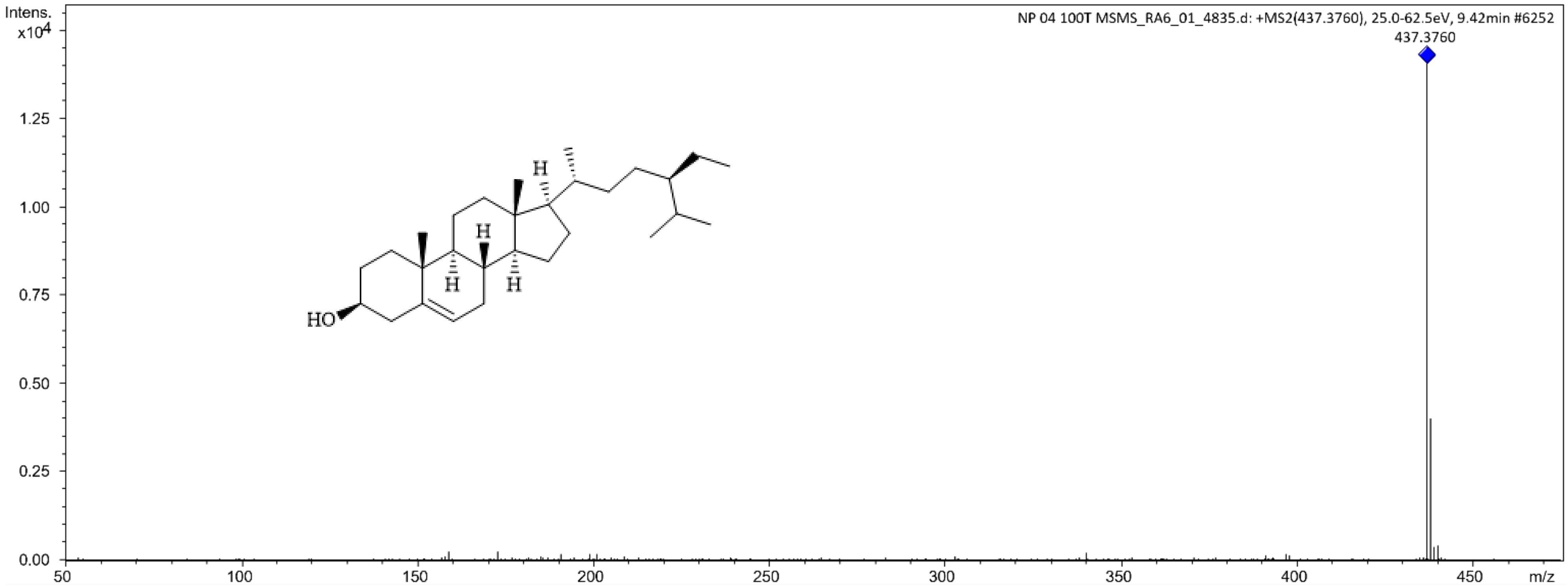

SUPPLEMENTARY FIGURE. S28. MS spectrum of β -Sitosterol.

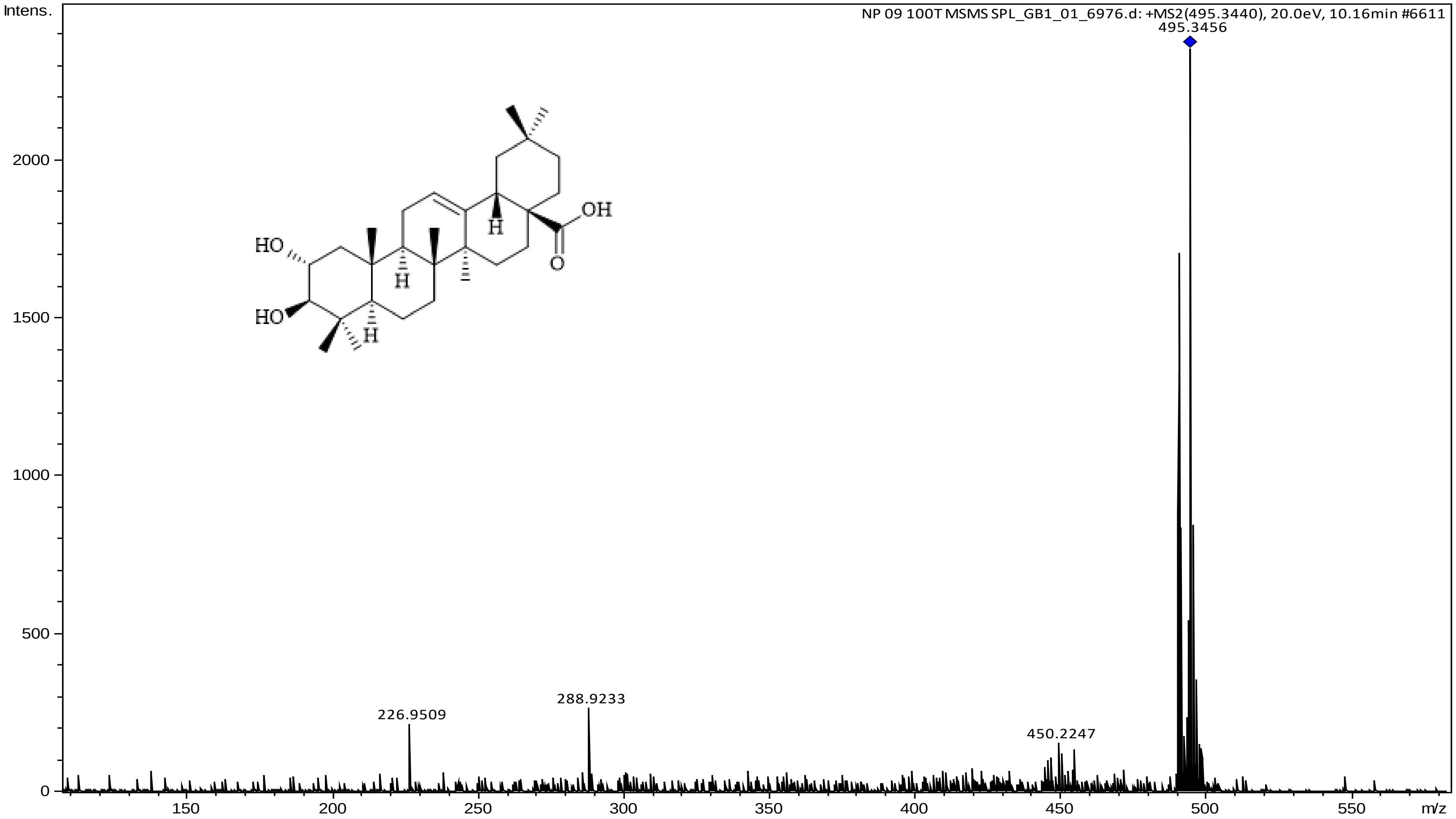

SUPPLEMENTARY FIGURE. S29. MS spectrum of maslinic acid.

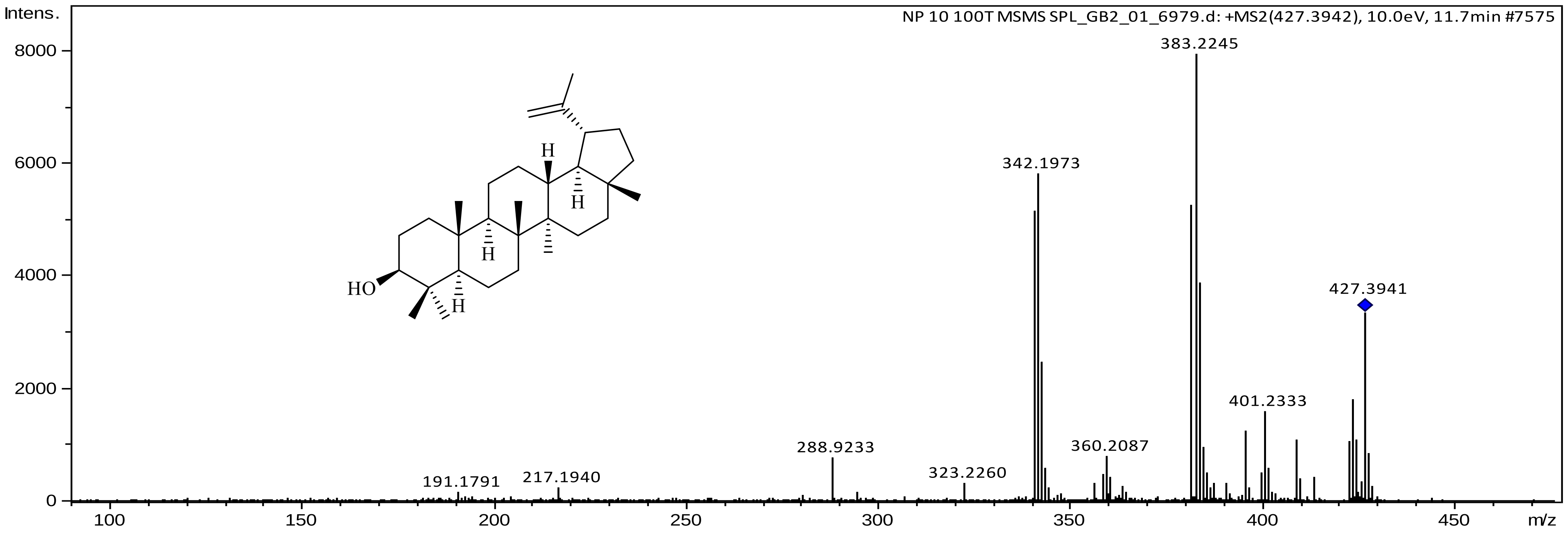

SUPPLEMENTARY FIGURE. S30. MS spectrum of lupeol.

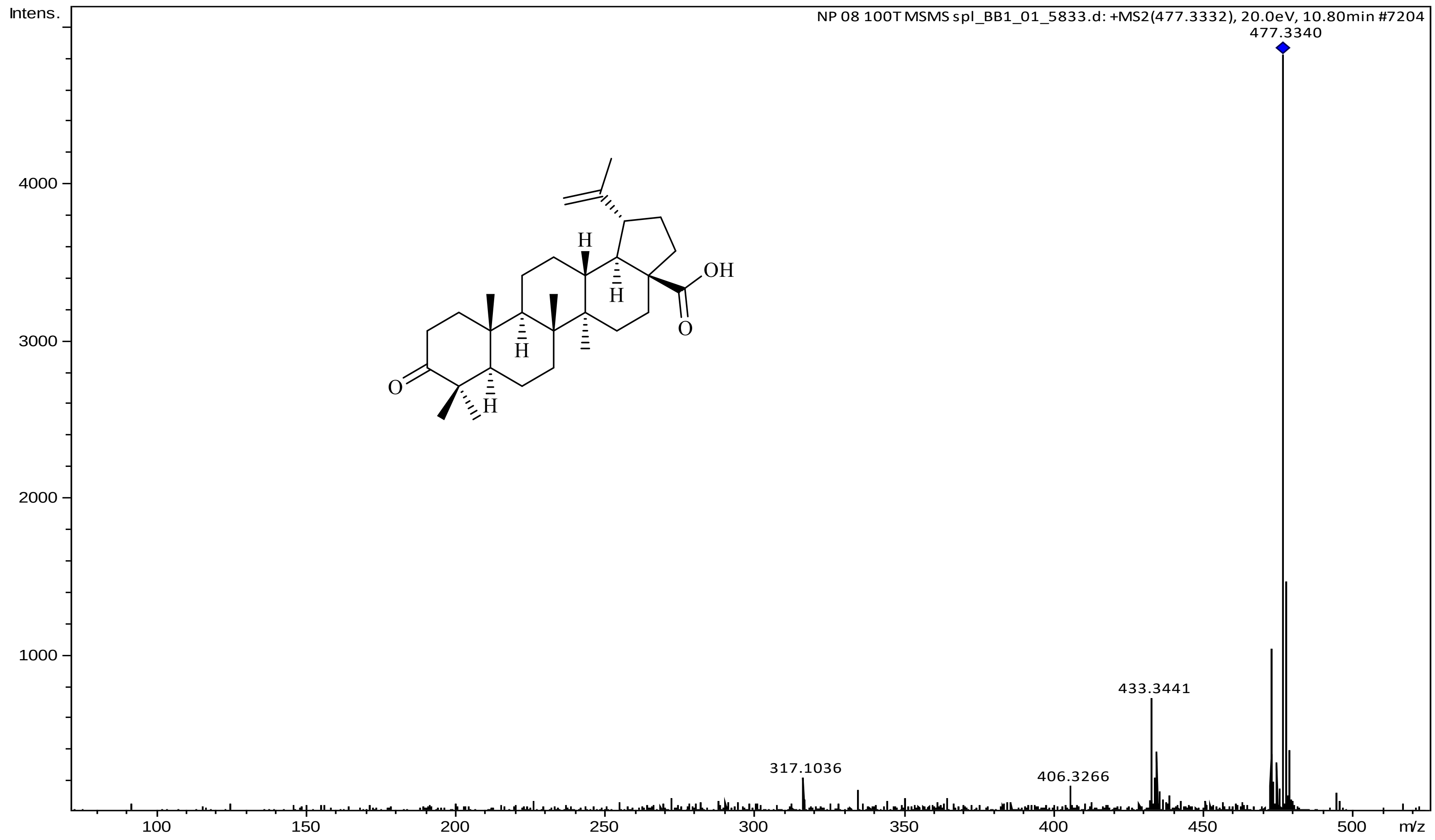

SUPPLEMENTARY FIGURE. S31. MS spectrum of betulonic acid.

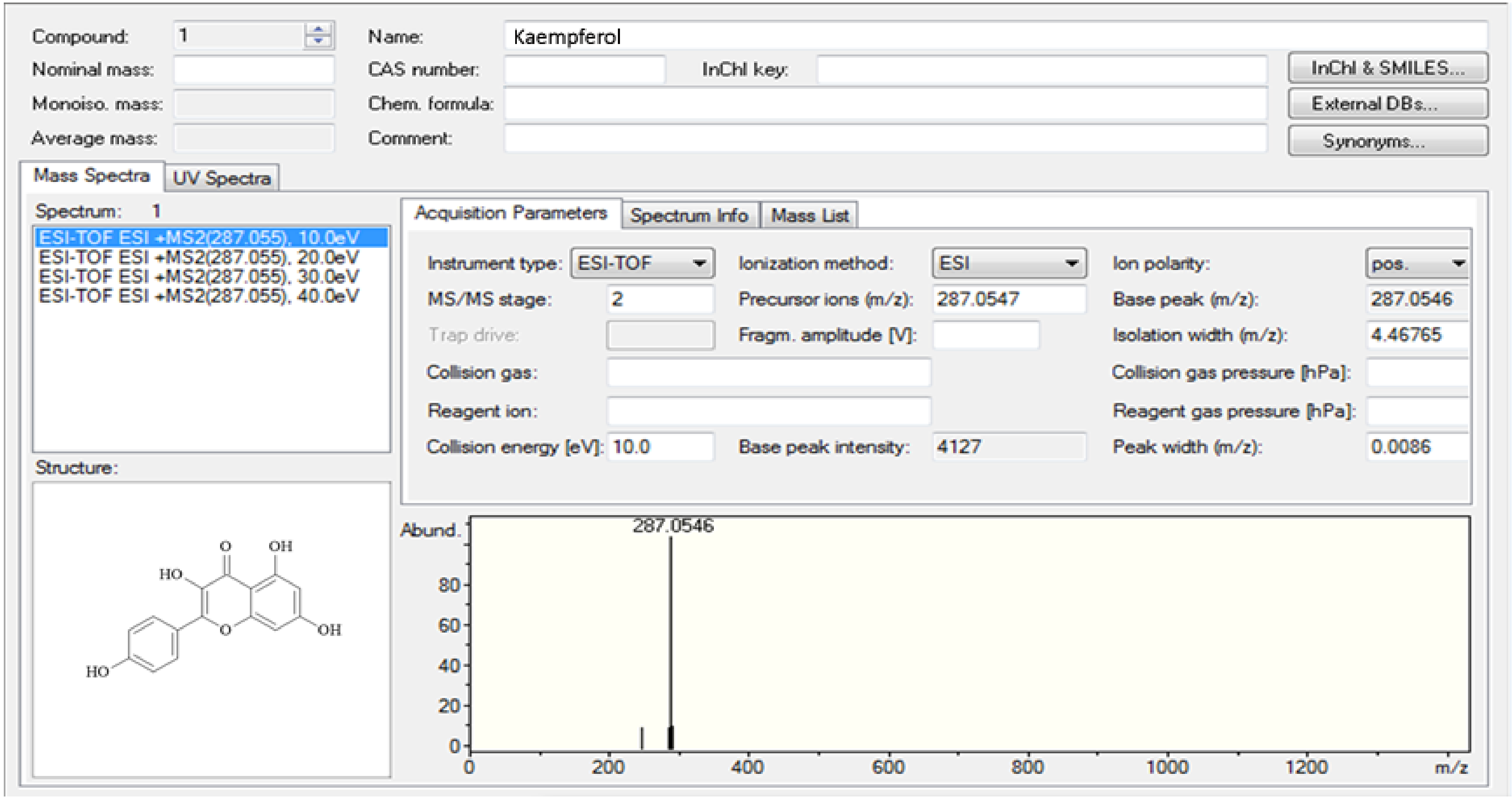

SUPPLEMENTARY FIGURE. S32. Depiction of kaempferol library record in Bruker Library Editor 4.4.

Naringenin

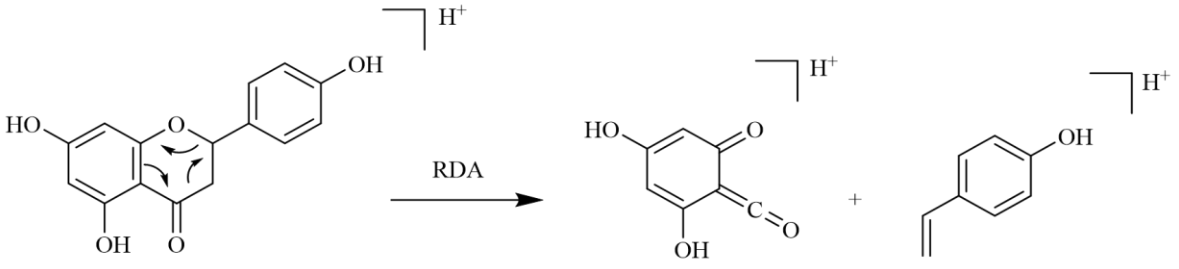

Chemical Formula: $C_{15}H_{13}O_5^+$
***m/z*: 273.0757**

Chemical Formula: $C_7H_5O_4^+$
***m/z*: 153.0182**

Chemical Formula: $C_8H_9O^+$
***m/z*: 121.0647**

Herniarin

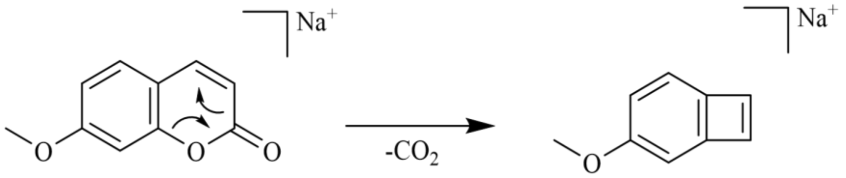

Chemical Formula: $C_{10}H_8NaO_3^+$
***m/z*: 199.0365**

Chemical Formula: $C_9H_8NaO^+$
***m/z*: 155.0467**

Oleanolic acid

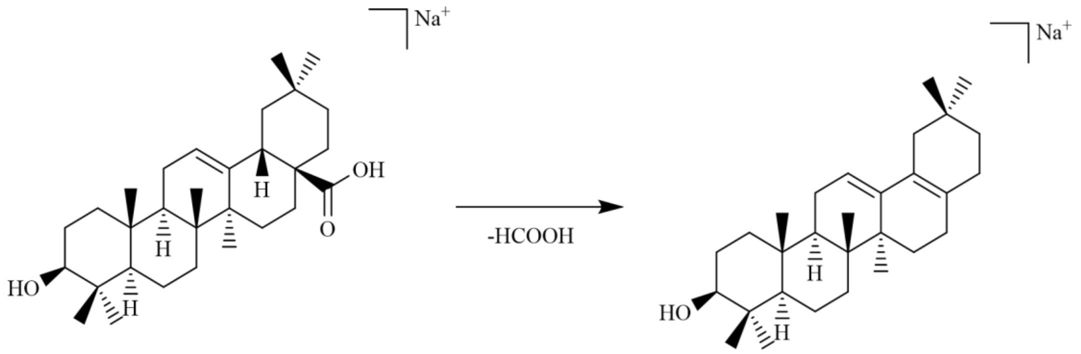

Chemical Formula: $C_{30}H_{48}NaO_3^+$
***m/z*: 479.3495**

Chemical Formula: $C_{29}H_{46}NaO^+$
***m/z*: 433.3440**

SUPPLEMENTARY FIGURE. S33. Depiction of the common fragmentation pathway of the investigated analyte.
